# Presentation of preload and afterload to isolated heart preparations

**DOI:** 10.1113/JP291198

**Published:** 2026-07-31

**Authors:** Esau Mhandu, Toan Pham, Kenneth Tran, Andrew J. Taberner, June‐Chiew Han

**Affiliations:** ^1^ Auckland Bioengineering Institute The University of Auckland Auckland New Zealand; ^2^ Department of Engineering Science and Biomedical Engineering The University of Auckland Auckland New Zealand

**Keywords:** *ex vivo* heart, load, Windkessel loading, working heart

## Abstract

The isolated heart preparation has been the fundamental model for performing mechanical and energetics investigations of cardiac function. Numerous reviews have been conducted detailing the evolution and development of Langendorff‐heart and working‐heart models, as well as considerations of experimental factors, including perfusion techniques, experimental conditions and applications in both preclinical and clinical areas. In this review, we provide the first narration and collection of the various techniques applied to load isolated heart preparations. Specifically, we describe how preload and afterload are presented, controlled and changed, either physically or using software, to prescribe pressure or flow impedance to the isolated heart. We also discuss the modelling of *in vivo* haemodynamics and how these conditions can be visualised in a working‐heart model. We complete the review by offering future perspectives on developing working‐heart models that not only aim to address the limitations of the current set‐ups but also extend to a dynamic impedance afterload device that can adapt beat‐by‐beat impedance changes, mimicking the *in vivo* condition more accurately.

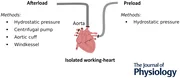

## Graphical abstract

**Abstract figure legend** The heart can be isolated, and when this is so, it must be subjected to loading conditions. Over the past few decades, various loading methods have been developed to prescribe preload and afterload to isolated working‐heart preparations. These include implementing physical and software‐based models to change the afterload of the working heart using hydrostatic pressure, by controlling pressure using centrifugal pump or aortic cuff or by controlling impedance within a Windkessel model. Preload has only been changed by applying hydrostatic pressure. As experimental cardiac physiology increasingly relies on precise, reproducible loading conditions to interpret contractile performance, energetics and translational relevance, we prepare this review to provide a comprehensive review of the implementation of preload and afterload to load isolated hearts and offer forward‐looking perspectives on next‐generation working‐heart systems.

## Introduction

An isolated heart is a preparation where the heart of an animal is dissected and removed from the body for its function to be studied, *ex vivo* (Grover & Singh, 2007; Watanabe & Okada, 2018). Thus, an isolated heart preparation is devoid of systemic circulation and neuromodulation. The use of isolated hearts in experiments has played a crucial role in studying and observing the physiological and pharmacological responses of cardiovascular parameters to various stimuli.

The aims and challenges in implementing this procedure are to maintain heart function when isolated from the body and to measure heart function under cardiovascular loads that reflect physiological loading conditions. Thus, in addition to the requirements of retrograde (Langendorff) perfusion, the isolated heart is often subjected to preload and afterload. Loading an isolated heart, a prerequisite for working‐heart mode, involves imposing preloads and afterloads that mimic those the heart would typically experience *in vivo*. Direct and independent manipulation of preload and afterload is important given their critical roles in determining myocardial function.

The isolated‐heart preparation was first demonstrated by Carl Ludwig and Elias Cyon in 1866 using the frog heart (Cyon, 1866) and later reviewed by Zimmer (1998). Oskar Langendorff, in 1895, extended the isolated‐heart preparation to mammalian hearts (cat, rabbit and dog) using a technique now well known as the Langendorff perfusion model (Langendorff, 1895). Since then isolated mammalian heart preparations have been extended to the working‐heart model, first introduced by James Neely and Howard Morgan in 1967 using the rat heart (Neely et al., 1967). Given its isovolumic nature and lack of change of afterload that inherently limit the physiological inferences that can be drawn, the working heart allows investigators to answer different types of questions that the Langendorff cannot. In the working‐heart mode, the perfusion pathway mimics *in vivo* circulation, where the perfusate is supplied to the left atrium, from which it flows into the left ventricle, ejects into the aorta and perfuses the coronary vasculature. A detailed history of the evolution and technical advancement of the isolated heart preparation has been covered previously (Leivaditis et al., 2024; Merin, 1988; Olejnickova et al., 2015; Taegtmeyer et al., 2016; Zimmer, 1998). Considerations of experimental conditions, including temperature, pacing, perfusate composition and heart viability criteria, have also been covered in detail (Bell et al., 2011; Hwang et al., 2010; Liao et al., 2012; Skrzypiec‐Spring et al., 2007; Sutherland & Hearse, 2000; Yeo et al., 2017). Current applications and potential perspectives of the isolated‐heart model in experimental research and clinical settings have also been reviewed (Leivaditis et al., 2024). These include integration of high‐resolution imaging techniques, advanced genomic and proteomic analyses, precision medicine, stem cell therapies, automation and artificial intelligence.

Over the past few decades loading methods have been developed, including using a balloon to change the preload of the Langendorff‐perfused heart (Obata & Takaki, 2018; Watanabe & Okada, 2018), implementing physical and software‐based models to change the afterload of the working heart using hydrostatic pressure (Forty et al., 1993; Goo, 2013; Goo et al., 2013; Goo et al., 2014a; Han et al., 2014; Han et al., 2018), controlling pressure (Schechter et al., 2014) or controlling impedance (Crystal et al., 2019; Elzinga & Westerhof, 1973; Howard & Iaizzo, 2019; Ishide et al., 1980; Westerhof et al., 1971). Impedance describes the opposition to the flow of blood in the arterial system and is the ratio between the time‐varying blood pressure and flow rate. In the intact circulation ventricular afterload is most comprehensively described by arterial impedance, which incorporates both the steady (resistive) and pulsatile components (compliant and inertial) of vascular opposition to flow (Garrett et al., 2019; Milnor, 1975). However, researchers when studying isolated heart systems re‐create afterload using a range of simplified or physiologically complete approaches that capture different elements of this impedance. In this review, we synthesise these developments and therefore provide the first overview of the various methods used to prescribe preload and afterload in isolated heart preparations, with particular attention to how these approaches approximate the physiological afterload defined by arterial impedance. Because impedance represents the most complete description of afterload in intact circulation, we highlight how different loading strategies capture distinct components of this physiological load. The way preload and afterload are applied to an isolated heart directly influences measurements of cardiac performance, as loading conditions strongly influence cardiac function, metabolism and experimental outcomes. Unlike the intact circulation, isolated‐heart systems must artificially re‐create load, and different methods can produce physiologically distinct states. A clear understanding of these available methods is critical for the isolated‐heart community, as it provides guidance on mechanical principles, methodological choices and their implications for supporting a more consistent, physiologically meaningful experimental design.

A central aim of this review is to examine how preload and afterload can be prescribed in isolated‐heart experiments to reproduce physiological loading conditions. In this review, preload or afterload is treated as an independent variable, that is, an experimentally controlled input used to simulate the mechanical loading environment of the intact circulation. The dependent variables of interest typically include ventricular or aortic pressure generation, stroke volume, cardiac output, oxygen consumption and coronary flow. Controlled preload and afterload are essential for mechanistic studies as they allow investigators to explore wide ranges of loading conditions on myocardial function and assess how the diseased heart responds acutely to physiological *versus* pathological loads, such as those arising from hypertension or arterial stiffening. Furthermore, the ability to prescribe preload and afterload enables the investigation of the chronic consequences of sustained loading changes or pharmacological testing and supports tissue and organ engineering platforms that require physiologically realistic mechanical loading environments.

## Perfusing the isolated heart

Perfusate is a fluid containing nutrients and oxygen to sustain the beating heart. Hormones can be added depending on the experimental design. Various methods have been used to perfuse an isolated heart. An early technique of perfusing the isolated heart involved using a donor or support animal. In this approach the excised heart of an animal is supported by perfusion with blood from another animal (donor) (Maughan et al., 1984; Obata & Takaki, 2018; Pasini et al., 1999; Suga & Sagawa, 1974; Sutherland & Hearse, 2000). In this method, the donor animal is anaesthetised, and its femoral artery and vein are cannulated. The arterial line is then connected to the cannulated aortic line of the isolated heart, and the effluent from the isolated heart is pumped back into the venous system of the donor animal for reoxygenation. Although this method has the advantage of supplementing real blood to the isolated heart, it has one drawback: any substance injected into the isolated heart is also transported to the support animal, which can result in adverse effects on the support animal. Additionally, the viability of the isolated heart is dependent on the survival of the support animal. Therefore, the deterioration of the support animal can threaten the viability of the isolated heart.

An alternative technique that does not require a donor animal is the replacement of whole blood with red blood cell–based perfusate, in which washed erythrocytes are suspended in a physiological buffer to restore plasma (Schechter et al., 2014; Sutherland & Hearse, 2000). This approach preserves the oxygen‐carrying capacity of blood while avoiding the need for a support animal. Although it has the same advantage as a support animal method, preparing washed blood can be labour‐intensive, requiring multiple centrifugation and washing steps to remove plasma proteins, leukocytes and platelets. This process not only increases the cost and time required for each experiment but also introduces variability depending on the source and handling of the blood. To avoid the need for erythrocytes altogether, several groups have instead used artificial oxygen carriers, such as haemoglobin‐based oxygen transport solutions, which provide oxygen delivery without the complexity of blood processing (Wrobeln et al., 2017).

The most common method of perfusing an isolated heart is to use a prepared artificial perfusate as a salt solution. A common perfusate solution is oxygenated ‘Tyrode's solution’ that typically contains, in mmol $L^{-1}$, 130 NaCl, 6 KCl, 1 Mg $Cl_{2}$, 1.5 Ca $Cl_{2}$, 0.5 Na $H_{2}PO_{4}$, 10 HEPES and 10 glucose (Goo, 2013; Goo et al., 2014b; Han et al., 2018). The solution is adjusted to pH 7.4 with Tris. Another common perfusate is a bicarbonate perfusion fluid as described by Krebs and Henseleit (Langendorff, 1895) with the following typical composition, in mmol $L^{-1}$: 118.5 NaCl, 25.0 Na $\mathrm{HC}O_{3}$, 4.7 KCl, 1.2 Mg $SO_{4}$, 1.2 K $H_{2}PO_{4}$, 11 glucose and 1.5 Ca $Cl_{2}$ with a pH of 7.4. The concentrations of ions can be modified to align with different experimental goals (Watanabe & Okada, 2018), for example high K^+^ (5× higher) to arrest the heart for measurement of basal oxygen consumption (Goo et al., 2014b; Han et al., 2014), and varying concentrations (Reichelt et al., 2009) of CaCl_2_.

## Isolated perfusion heart models across species

Hearts from mammalian (cat (Gabel et al., 1966; Vogel & Lucchesi, 1980), rabbit (Caldwell et al., 2012; Elliott et al., 2012; Forty et al., 1993; Kronsteiner et al., 2023; Murphy et al., 2017; Wang et al., 2014), dog (Ishide et al., 1980; Nishioka et al., 1987; Sunagawa et al., 1982), pig (Brook et al., 2020; Gellner et al., 2020; Howard & Iaizzo, 2019; Kappler et al., 2019; Pigot, Steen et al., 2024), rat (Günzinger et al., 2007; Neto‐Neves et al., 2017), mouse (Ackers‐Johnson et al., 2016; Eberli et al., 1998; Li et al., 2020), human (Brook et al., 2020; Nanthakumar et al., 2007)) and non‐mammalian (frog (Zimmer, 1998, 1999), chicken (Hess et al., 2020; Wack & Hamlin, 1986), trout (Klaiman et al., 2014)) species have been used in isolated perfusion modes. Isolated perfusion of large‐animal hearts, such as pig and dog, has been performed less frequently due to the high cost of the procedure, high complexity, large volumes of perfusate required and more extensive equipment requirements (Bell et al., 2011; Colah et al., 2012; Skrzypiec‐Spring et al., 2007; Sutherland & Hearse, 2000; Ytrehus, 2000). Isolated human hearts have also been used (Brook et al., 2020; Nanthakumar et al., 2007), but their use is limited by challenges in procuring and maintaining these samples; most human hearts are acquired as explanted donor hearts that are no longer suitable for transplantation (Brook et al., 2020). In contrast, the rodent heart has been the most commonly used preparation for Langendorff and working‐heart perfusion experiments because of its small size, ease of handling and highly replicable performance (Grieve et al., 2004; Sutherland & Hearse, 2000; Ytrehus, 2000).

Although isolated perfusion has been applied across a wide range of species, the composition and physiological relevance of commonly used saline‐based perfusates differ substantially between them. Rodents, for example, tolerate crystalloid solutions with supraphysiological glucose concentrations rather than normal glucose levels to sustain cardiac function (Liao et al., 2012; Neely et al., 1967; Skrzypiec‐Spring et al., 2007; Sutherland & Hearse, 2000). Although fatty acids are the main energy source *in vivo*, they are rarely included in isolated heart preparations because of their poor solubility in aqueous solutions and the resulting frothing (Bell et al., 2011; Liao et al., 2012; Sutherland & Hearse, 2000). As a result, most crystalloid perfusates rely on elevated glucose concentrations as the primary metabolic substrate. The same solubility constraints also limit the use of albumin in perfusion solutions. The absence of albumin in standard crystalloid media eliminates colloid osmotic pressure, resulting in myocardial oedema and impaired coronary perfusion (Bell et al., 2011; Hwang et al., 2010; Pigot, Soltesz et al., 2024; Sutherland & Hearse, 2000; Usai et al., 2025). Furthermore, saline‐based perfusates lack physiological viscosity, producing higher shear stress than would occur *in vivo*, altering coronary resistance (Skrzypiec‐Spring et al., 2007). To partially compensate for these limitations, many preparations supplement the perfusate with pyruvate, which improves mitochondrial redox balance, enhances oxidative ATP production and augments contractile recovery across species (Cai et al., 2025; Grieve et al., 2004; Hwang et al., 2010; Liao et al., 2012; Randle et al., 1964).

## Langendorff technique: preload

Various definitions of preload have been used in the literature. At the tissue level, preload is typically defined as the strain imposed on myocardial fibres in the relaxed state (Ascenzi & Kane, 2007; Collins & Dias, 2014; Nguyen et al., 2018; Strafford, 2006). The fibre length refers to the end‐diastolic sarcomere length or muscle length of the myocardium, which can be quantified in isolated muscle preparations. At the whole‐heart level, preload is typically defined as the end‐diastolic volume of the left ventricle when it is filled with blood (Moore, 2002). In all these definitions, muscle end‐diastolic length or left‐ventricular end‐diastolic volume before the beginning of systole is used as a measure of preload (Eskander & Kern, 2017; Moore, 2002; Pagel & Freed, 2018; Usai et al., 2025). Preload has also been defined to reflect the force that stretches the muscle during diastole (Little & Little, 1982). Thus, it reflects the initial pressure of the left ventricle at the end of diastole prior to contraction (Little & Little, 1982; O'Keefe & Singh, 2025). Minority has taken preload as defined (Luecke et al., 2004) or indicated (Diebel et al., 1992; Michard et al., 2003) by fibre length or ventricular volume during diastole.

**Figure 1:**
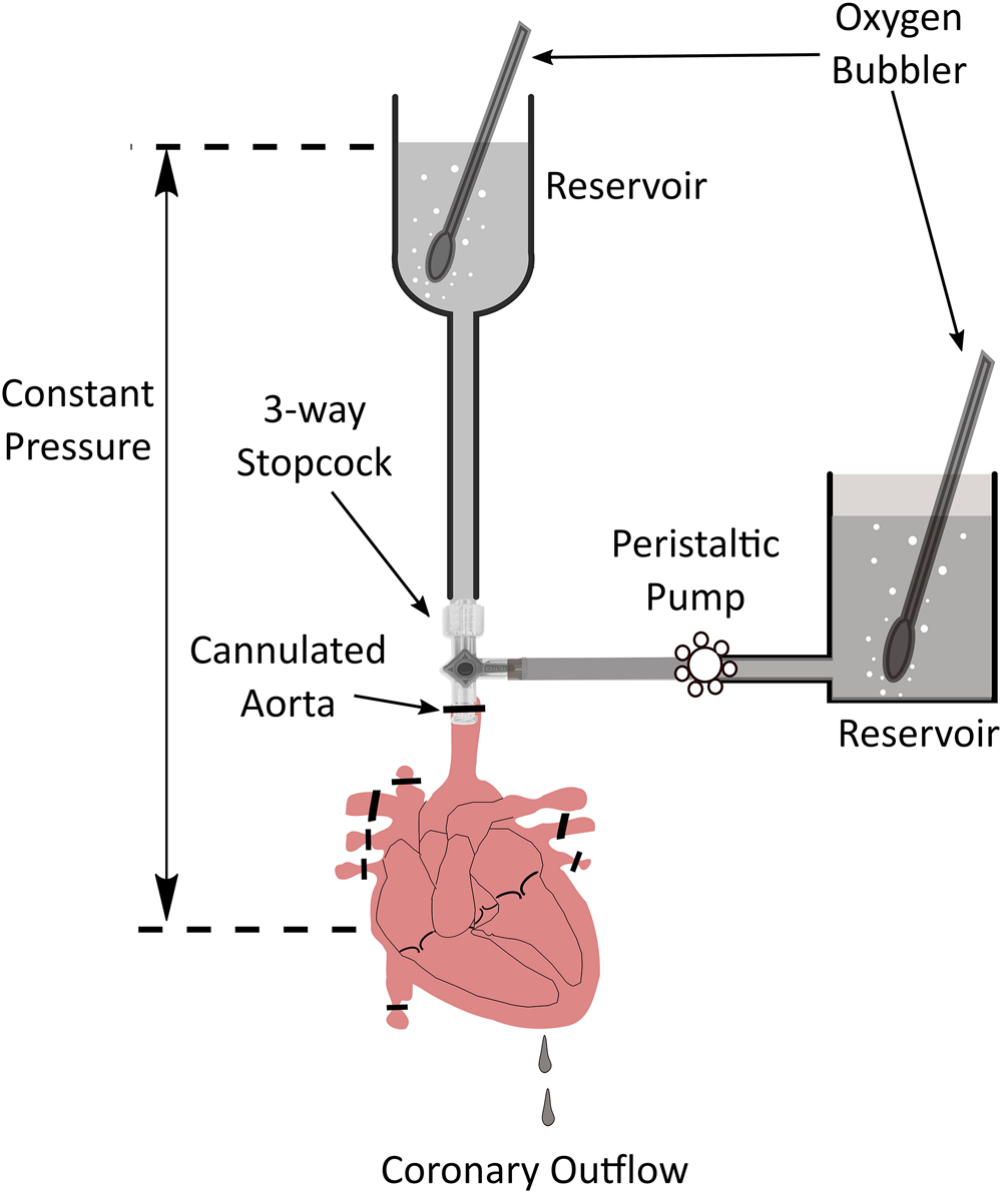
Langendorff perfusion: constant‐pressure mode versus constant‐flow mode Two common methods of Langendorff‐perfusing are used for an isolated heart: (1) constant‐pressure mode, achieved by maintaining the perfusate reservoir at a fixed height and (2) constant‐flow mode, achieved by setting the peristaltic pump to a constant speed. The three‐way stopcock allows switching between these two modes of Langendorff perfusion. Perfusion pressure can be measured with a catheter inserted into the cannulated aorta, whereas flow rate can be measured with a flow sensor placed in the aortic line. In addition to the cannulated ascending aorta all other vessels can be ligated except for the pulmonary artery, which is kept open to allow coronary effluent to exit and be collected.

In the Langendorff technique, an isolated heart is retrogradely perfused via the cannulated ascending aorta. The aortic valve remains closed under pressure, and the perfusate passes through the coronary arteries via coronary ostia (Langendorff, 1895), from where it is drained off to the coronary sinus and into the right atrium via the coronary veins. The Langendorff‐perfused heart can be perfused at either constant pressure or constant flow. The former perfusion mode is typically achieved by maintaining a constant hydrostatic pressure through setting the perfusate reservoir at a fixed height above the heart. The constant perfusion pressure is usually in the range of 50  to 70 mmHg in isolated rat heart models (Cai et al., 2025; Hwang et al., 2010). In the latter perfusion mode, constant flow is achieved using an infusion or roller pump that sets a constant perfusion rate (Cai et al., 2025; Dhein, 2005, 2005; Hatami et al., 2020; Pigot et al., 2022; Sutherland & Hearse, 2000; Sutherland et al., 2003). A set‐up that enables switching between the two perfusion modes is shown in Fig. 1.

Both Langendorff perfusion modes described in Fig. 1 present neither preload nor afterload to the isolated heart. Because perfusate is delivered retrogradely through the aorta, the aortic valve remains closed, preventing ventricular filling and ejection. Essentially, the Langendorff‐perfused heart can only contract isovolumetrically.

**Figure 2:**
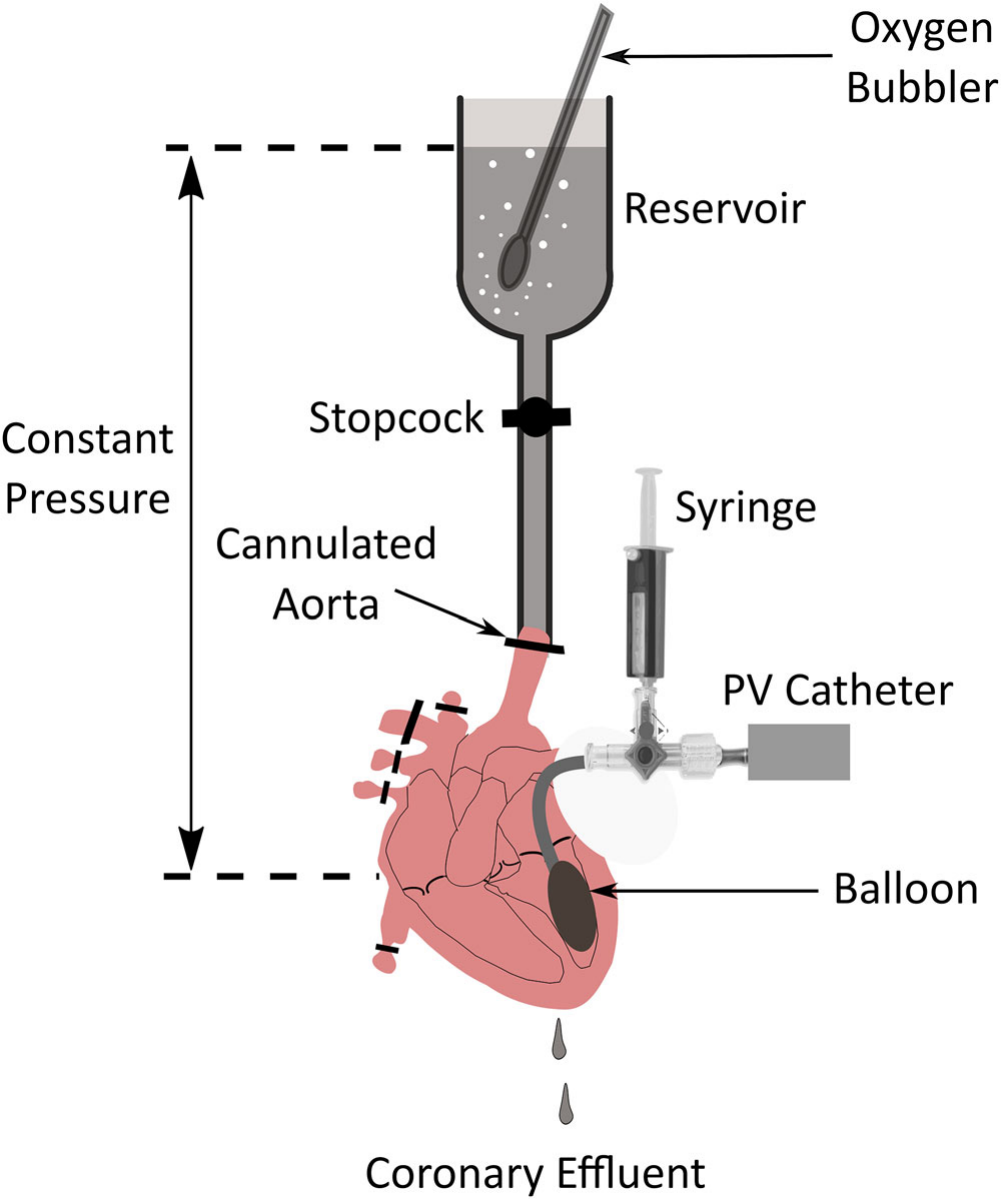
A balloon inserted into the left ventricle to prescribe different ranges of preload to a Langendorff‐perfused heart The heart can only undergo isovolumic contractions in this system because the left ventricle contracts without ejecting fluid. A portion of the left atrium is dissected from the isolated heart to expose the ventricular inlet for balloon insertion. A pressure–volume (PV) catheter is inserted into the balloon to measure the pressure–volume changes at each preload level. A syringe is used to increase the left‐ventricular volume by inflating the balloon.

In the Langendorff‐perfused heart, preload can be imposed and adjusted using a balloon inserted into the left ventricle (Bell et al., 2011; Obata & Takaki, 2018; Usai et al., 2025; Watanabe & Okada, 2018) via the left atrium (Fig. 2). The balloon is typically made of a thin, compliant material, for example latex (Obata & Takaki, 2018), combined with a plastic wrapping film (Curtis et al., 1986; Sutherland et al., 2003). To insert the balloon into the left ventricle, the left atrial appendage is typically removed. The balloon is filled with water and is connected to a pressure transducer and a syringe. The balloon's size is manipulated by inflating or adjusting the balloon's water volume to set and vary diastolic pressure and, therefore, preload. Because the balloon is sealed and prevents outflow, the left ventricle undergoes pressure changes during systole and diastole at fixed volumes (isovolumetric contraction and relaxation). Thus, in this set‐up, isovolumic systolic and diastolic left‐ventricular pressures can be measured over a range of volumes (or preloads).

The balloon insertion technique is typically used for the left ventricle, but a similar approach has been extended to the right ventricle and applied to study hearts from other species, including the mouse (Ackers‐Johnson et al., 2016; Eberli et al., 1998; Li et al., 2020), rat (Neto‐Neves et al., 2017), rabbit (Caldwell et al., 2012; Murphy et al., 2017; Wang et al., 2014), chicken (Hess et al., 2020; Wack & Hamlin, 1986) and rainbow trout (Klaiman et al., 2014).

## Working heart: preload

Unlike in Langendorff mode, in which the heart is perfused retrogradely and the perfusate neither enters nor is ejected from the left ventricle, an isolated working‐heart model involves physiologically filling and ejection from the left ventricle. During diastole, perfusate flows into the left atrium and passes into the left ventricle. During systole, it is then ejected into the aorta. The ejected perfusate subsequently perfuses the myocardium via coronary ostia, particularly when the aortic valve closes following ejection. In the working‐heart, as in the Langendorff preparation, coronary perfusion pressure is determined by the hydrostatic head of the reservoir position above the heart. The reservoir is adjusted to maintain a pressure typically between 30 and 100 mmHg, approximating the systolic afterload (as described in the next section). This pressure gradient drives coronary perfusion.

**Figure 3:**
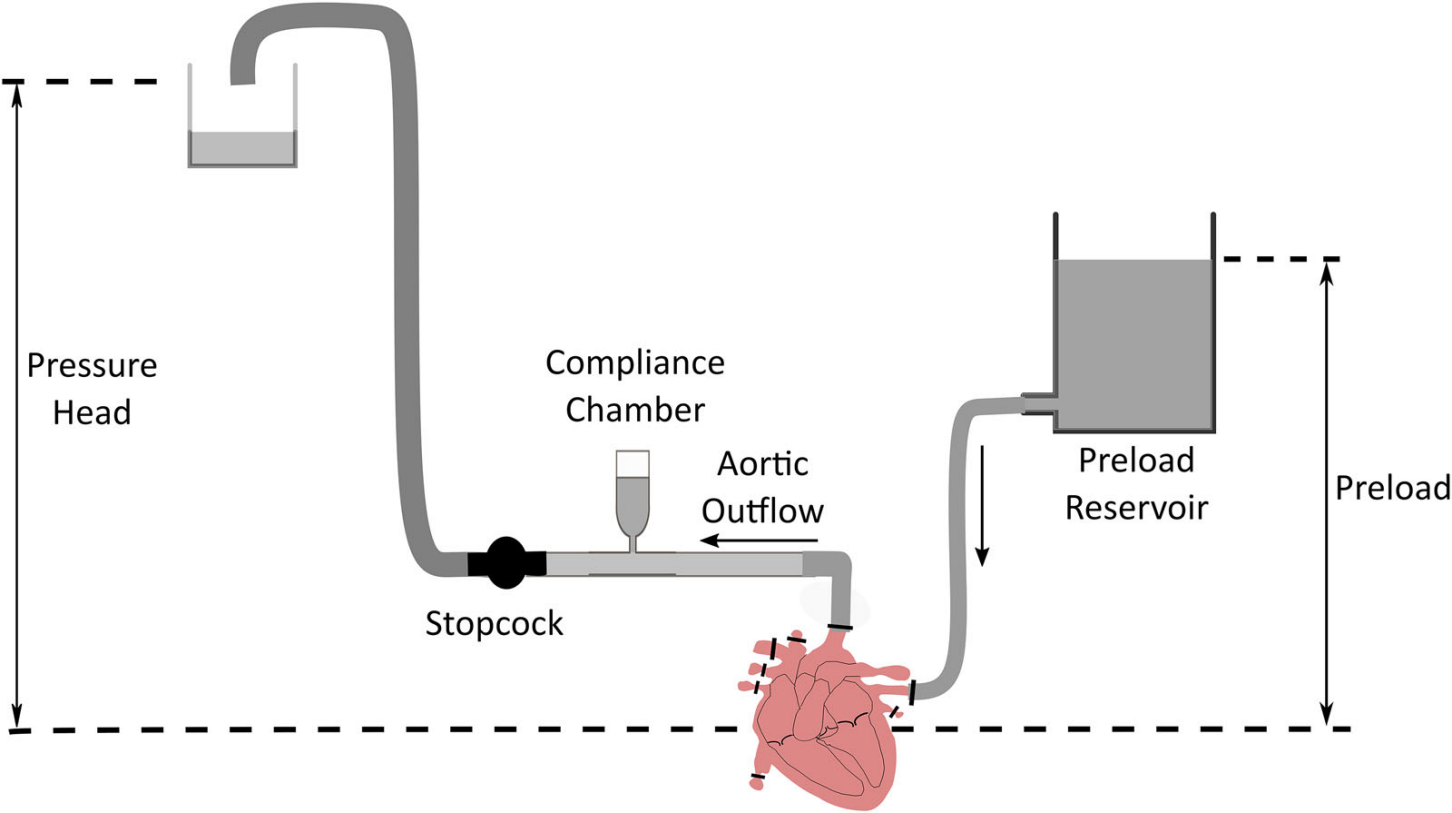
Isolated working‐heart system set‐up The preload reservoir is bubbled with oxygen (not shown). The preload is kept constant by keeping the preload reservoir at a fixed height. The afterload driving against the heart comprises a compliance chamber, a resistance element (stopcock) and a hydrostatic pressure head. The system uses a stopcock to model the peripheral resistance and a compliance chamber to model the compliance of the aorta. The arrows indicate the direction of flow of the perfusate entering the left ventricle and exiting through the aorta. The figure is drawn based on the concept introduced by Otto Frank (Frank, 1959).

The preload used during working‐heart experiments typically ranges from 5 to 20 mmHg (Goo et al., 2014b, 2014a). The preload determines the pressure and, therefore, the volume of perfusate filling the ventricle during diastole and is adjusted by varying the height of the preload chamber. As with the Langendorff preparation either constant‐pressure or constant‐flow modes can be applied to the isolated working heart. In the constant‐pressure mode, the preload chamber and the perfusate meniscus are maintained at a fixed height above the heart, generating a hydrostatic pressure that drives perfusate into the left atrium. When using a donor animal to support an isolated working heart, the excised heart and the support animal are positioned at the same level to maintain this hydrostatic pressure gradient (Suga & Sagawa, 1974). In the constant‐flow mode, a flow‐regulating pump (e.g. a peristaltic or roller pump) replaces the hydrostatic pressure head, delivering perfusate at a set flow rate (Gorski et al., 2018; Hatami et al., 2020; Plačkić & Kockskämper, 2018). The pump's output (flow rate) can be precisely adjusted via hardware or software controls to meet experimental requirements. Between these methods, constant‐pressure preload control is more widely adopted due to its relative simplicity of set‐up and operation. Detailed schematics of working‐heart preparation can be found elsewhere (DeWitt et al., 2016; Gross, 2009; Loiselle et al., 2014; Pham et al., 2018; Ruiz et al., 2015; Yang et al., 2024) and are summarised in Fig. 3.

The earliest innovation in the isolated working mammalian heart was achieved by Henry Newell Martin (1881) through the development of the mammalian heart–lung preparation using cats and dogs (Martin, 1881, 1883). He realised the importance of maintaining the coronary circulation and observed that the isolation techniques used for the hearts of cold‐blooded animals did not apply to mammals. In his set‐up, the entire dog's body was placed in the observation chamber; that is, the heart was not completely isolated from the rest of the body. All major vessels leading to and from the heart were ligated, leaving only the pulmonary circulation intact. This ‘*physiological isolation*’ allowed the lungs to provide pulmonary circulation and artificial respiration. The blood pathway in his preparation was ‘*left auricles, left ventricle, aortic arch … the coronary vessels, the right auricle, the right ventricle, the pulmonary circulation … and back to the left auricle*’. The dog's body was maintained at a constant temperature by heating water beneath the chamber with burners. The heart was perfused with defibrinated blood from other dogs, which was whipped and strained to prevent clotting. Under these conditions the heart continued to beat for up to 5 h.

Although preload and afterload were not systematically manipulated, Martin noted that venous pressure (preload) could be adjusted by lowering flasks positioned in the chest (Martin, 1883). Similarly arterial pressure (afterload) could be altered by changing the height of the aortic tube. From this pioneering work, it became clear that both preload and afterload could be controlled by adjusting the height of reservoirs or components in the experimental apparatus.

Building on these foundational concepts, Otto Frank, a German physiologist, advanced the development of isolated heart systems in the late 1890s, enabling both preload and afterload to be systematically varied and controlled. Frank aimed to understand the complex interaction of the heart and the network of arteries and veins. Based on experiments with isolated frog hearts, he developed mathematical models to investigate how changes in vascular elasticity and resistance shaped the arterial pulse, as later summarised in modern analyses of his work (Frank, 1990; Kuhtz‐Buschbeck et al., 2018; Sagawa et al., 1990). In one of his earliest publications, translated in ‘On the dynamics of the Cardiac Muscle’ in 1895 (Frank, 1959), Frank introduced an isolated working‐heart frog model coupled to an artificial circulatory system (Fig. 3), enabling precise manipulation of vascular parameters. This set‐up laid the experimental foundation for understanding ventricular function under defined preload and afterload conditions.

**Figure 4:**
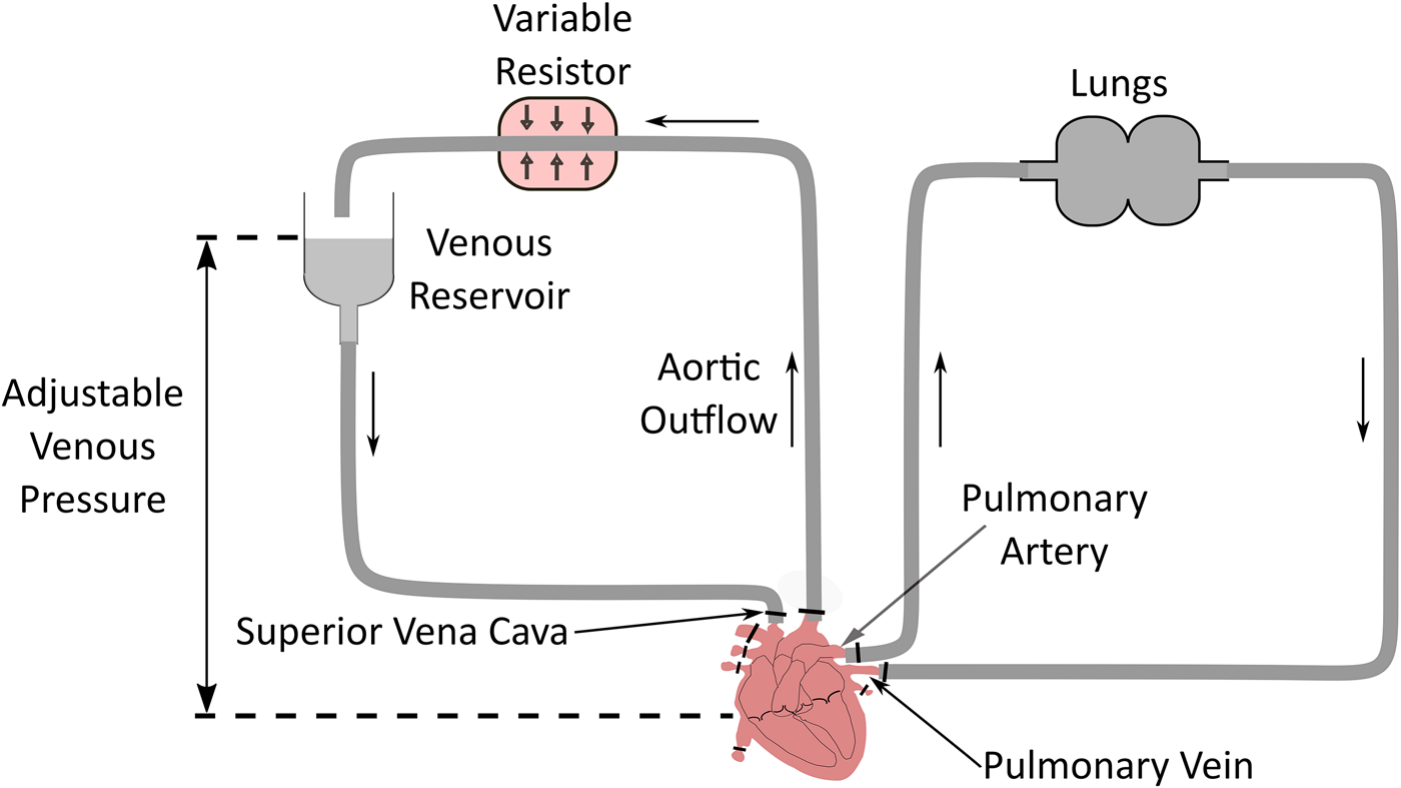
The improved heart–lung preparation set‐up by Starling The system implements a variable resistor that models the peripheral resistance of the systemic circulation, thereby setting the afterload. Venous pressure, and thereby the preload, is adjusted by the height of the venous reservoir. The lungs provide artificial ventilation. The major vessels, including the superior vena cava, pulmonary artery, pulmonary vein and aorta, are all cannulated. The arrows indicate the direction of flow of the perfusate. The illustration has been adapted from a figure by Starling (1930).

It is important to note that Frank's preparation was not a heart–lung system as developed by Martin (1881). Instead his approach involved perfusing the isolated frog heart with diluted mammalian blood, typically obtained from sheep or cattle, thereby achieving complete isolation from the pulmonary circuit. This methodological separation of the heart from the rest of the circulation presented a crucial step towards more advanced perfusion systems. Building upon Martin's original design (1881), Ernest Henry Starling, a British physiologist, later advanced the mammalian heart–lung preparation in 1912 using canine hearts and blood (Knowlton & Starling, 1912; Starling, 1930). One of Starling's many heart–lung preparations, as detailed in his textbook, *The Principles of Human Physiology (1930)* (Starling, 1930), is illustrated in Fig. 4. Whereas Martin's system relied on a hydrostatic pressure head to establish afterload, Starling's preparation introduced a controllable ‘variable resistor’ to modulate aortic flow and precisely adjust the afterload presented to the heart. This resistor consisted of a thin‐walled rubber tube enclosed in an air‐pressurised glass chamber; by increasing the surrounding air pressure, the tube was compressed, thereby increasing its resistance to blood flow.

**Figure 5:**
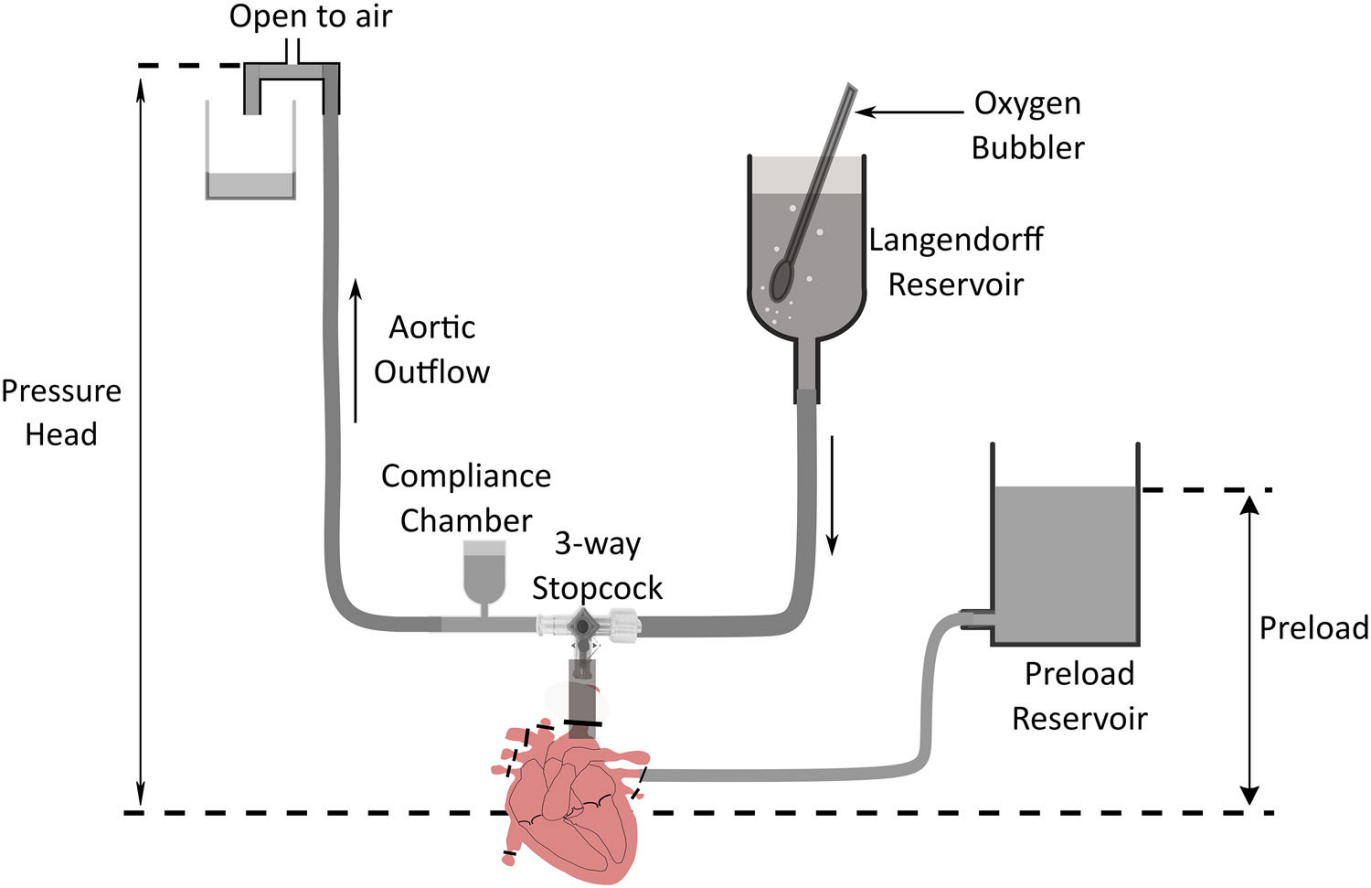
Isolating working heart developed by Neely and Morgan in 1967 Ventilation was achieved through the use of oxygenation chambers instead of isolated lungs. The compliance chamber was used to model the elasticity and distensibility of the aorta. The afterload was made up of a hydrostatic pressure head, which allowed it to be varied by adjusting the height of the pressure head with respect to the heart. This schematic is drawn based on a figure by Neely et al. (1967).

Further advancements were achieved by Neely and Morgan (Neely et al., 1967), who refined the isolated working‐heart model by replacing the lungs with an artificial respiration system (using an oxygenation reservoir). This refinement allowed direct control of left‐ventricular load while enabling both preload and afterload to be varied independently (Fig. 5).

Neely et al. (1967) devised a completely isolated rat heart preparation, where the lungs were removed and ‘perfusate was introduced into the left atrium at various filling pressures and pumped by the left ventricle against a hydrostatic pressure head’. Preload was varied by adjusting the height of the preload reservoir relative to the heart (Fig. 5). The model accounts for the elasticity of the aorta, that is the compliance of the aortic walls, using a compliance chamber filled with air. The volume of air in the chamber was used to determine the ‘size and shape of the aortic pressure curve.’ Neely et al. used this set‐up to investigate the effects of left‐atrial filling pressure (preload) on left‐ventricular pressure development and myocardial oxygen consumption. The height of the aortic column was kept fixed to provide a fixed hydrostatic afterload. Although afterload was not systematically investigated, aortic outflow resistance was modulated in some experiments by varying tubing diameter through manual compression and release of the tube. This is analogous to the variable resistor used by Starling (1930) (Starling, 1930) to modulate aortic impedance and thus alter afterload.

## Working heart: afterload

**Figure 6:**
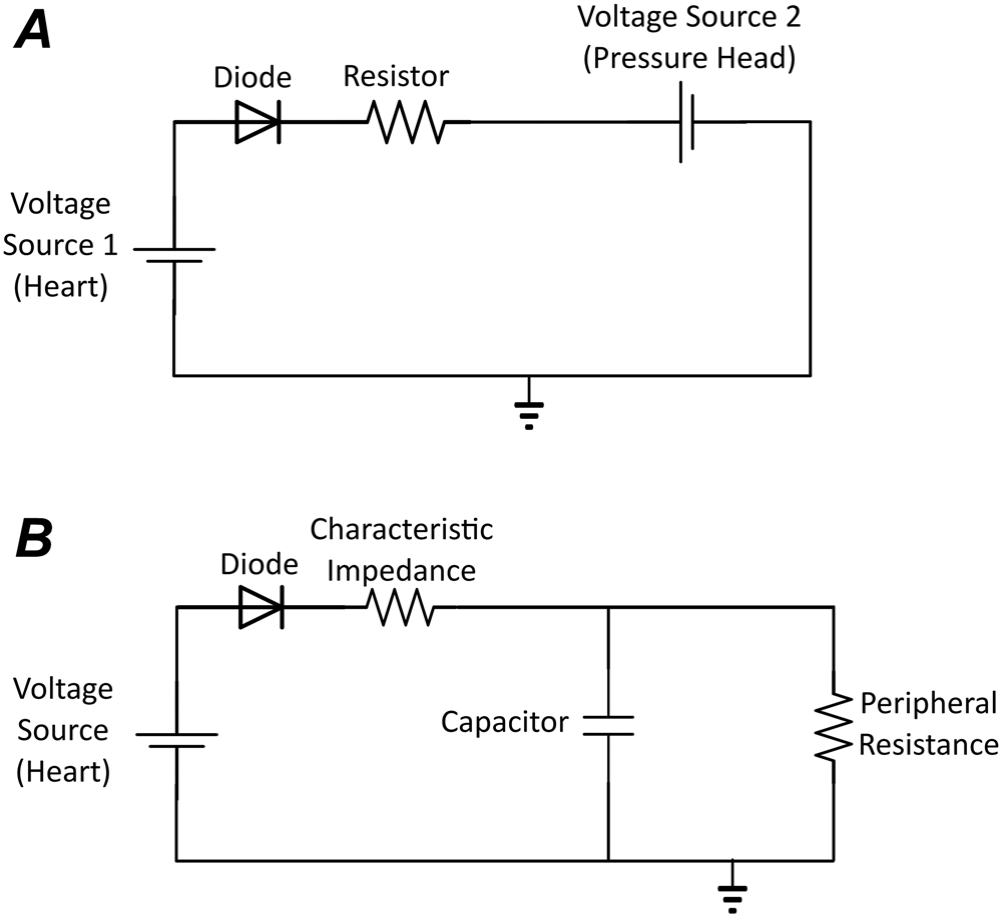
Electrical equivalence circuit diagrams for isolated working‐heart systems that represent afterload as a pressure (*A*) or as an impedance (*B*) *A*, the left ventricle is represented as a voltage source. The diode represents the aortic valve, which allows one‐directional flow of perfusate. The aorta provides resistance to the flow of perfusate, which is represented by a resistor. The afterload is represented as voltage source 2. *B*, the ejected perfusate is stored in the ‘aorta’ (tubing connected to the aorta) during systole, with a compliance represented by a capacitor and dissipated through the rest of the system (body) that has a peripheral resistance represented by the peripheral resistor. Afterload is thus characterised by the three variables (characteristic impedance, capacitor and peripheral resistance) which make up a three‐element Windkessel system.

For the heart to eject blood into the aorta, the pressure developed in the ventricles must exceed the aortic pressure, which constitutes the afterload. In an isolated working‐heart model, the left ventricle pumps against an afterload and can eject perfusate when the left‐ventricular pressure exceeds the set afterload. The precise definition of ‘afterload’ is thus critical for system design. Various terms and definitions have been used to describe afterload. Typically, afterload is characterised as the force (Westerhof et al., 1971; Schechter et al., 2014) or the pressure (Mellors et al., 2001; Iribe et al., 2007; Han et al., 2014; LaCombe et al., 2024, Anon, n.d.) that the heart must develop to eject blood during systole. Alternatively, afterload has also been conceptualised as the flow impedance presented by the arterial system (Milnor, 1975; Garrett et al., 2019; Pitoulis et al., 2022; Dowrick et al., 2022; Pigot et al., 2022, 2023). Milnor (1975) (Milnor, 1975) argued that such pressure‐based definitions are incomplete in the intact circulation because pressure and wall stress are manifestations of the load rather than the load itself. He proposed that the arterial impedance spectrum, which captures the resistive, compliant, inertial and wave‐reflection properties of the arterial tree, provides the only rigorous description of the external forces opposing ventricular ejection. In contrast, Noble (1979) distinguished between the load on the myocardium (wall stress), the load on the ventricle (ventricular pressure) and the load imposed by the arterial system (impedance). In Noble's framework, impedance determines the load, but it is not itself the load. He argued that the ventricle experiences afterload as mean left‐ventricular pressure because instantaneous or peak pressures are distorted by arterial compliance, inertance and valve dynamics. Together, these perspectives highlight that afterload can be conceptualised either as an external arterial load (impedance) or the internal left‐ventricular load (pressure), and that the choice of definition has practical consequences on the afterload model design. To illustrate the physiological differences between afterload as a fixed pressure *versus* as an impedance, an equivalent electrical circuit is presented in Fig. 6.

These differing definitions have led to distinct working‐heart model designs. Models adopting a fixed hydrostatic pressure as afterload produce pressure–volume loops with a characteristic constant pressure during ejection (i.e. flat‐top loops), as distinct from the curved morphology (i.e. time‐varying ejection pressure) typically observed *in vivo* during ejection (Khalafbeigui et al., 1979; Wu et al., 1989). In contrast, models defining afterload as vascular impedance, consistent with Milnor's (1975) (Milnor, 1975) framework, yield pressure–volume loops that more closely replicate the *in vivo* loop ejection curvature (Schechter et al., 2014). This distinction underscores the importance of selecting an afterload representation that aligns with the physiological phenomena a model aims to reproduce.

Further support for impedance‐based afterload modelling comes from *in vivo* studies demonstrating that the key Windkessel parameters, namely peripheral resistance (*R*_p_) and systemic arterial compliance (*C*), change on a beat‐to‐beat basis. One such study is from Molino et al. (Molino et al., 1998), who applied a two‐element Windkessel model to the ascending aortic pressure and flow recordings in conscious rats. Their findings showed that compliance and the time constant exhibit beat‐to‐beat variability, even under steady conditions, and respond appropriately to pharmacological interventions such as vasodilatation and vasoconstriction. These results reinforce the view that afterload is inherently dynamic, and simplified Windkessel models can capture physiologically meaningful changes in vascular properties that vary beat to beat. This provides experimental justification for implementing dynamic, impedance‐based afterload systems in isolated working‐heart preparations, particularly when the goal is to reproduce the time‐varying load experienced *in vivo*.

After the historical development of isolated working‐heart preparations and prescription of preload in the section above, the following sections review the evolution and implementation of various afterload control systems employed in these models. The afterload control system falls into three main categories: (1) hydrostatic pressure‐based systems, (2) variable pressure‐controlled systems and (3) variable resistance/impedance‐controlled systems.

The stability of isolated heart preparations does not seem to be vary substantially between modes of perfusion. Curtis et al. (1986) managed to sustain a Langendorff‐perfused heart for 4 h. The functionality of the working heart is preserved for 2 to 5 h (Martin, 1881; Neely et al., 1967; Elzinga & Westerhof, 1973; Duvelleroy et al., 1976; Olders et al., 1990; Segel & Follette, 1993). Although many studies report that isolated hearts can remain viable for several hours, most also note a progressive drift in performance over time (Sutherland et al., 2003; Bell et al., 2011; Usai et al., 2025). Contractile function typically deteriorates at a rate of 5% to 10% every hour (Sutherland & Hearse, 2000).

## Afterload – using hydrostatic pressure

Using a hydrostatic pressure involves creating a fixed pressure head against which the heart drives. An example is illustrated in Fig. 5, drawn based on the rat working‐heart system of Neely et al. (Neely et al., 1967), where afterload could be adjusted by varying the height of the aortic column. This loading approach, modulating afterload by changing the pressure head height, was subsequently adopted by researchers studying rabbit hearts (Forty et al., 1993) and diseased rat hearts (Goo, 2013; Goo et al., 2013, 2014a; Loiselle et al., 2014; Han et al., 2014, 2018).

Although afterload *in vivo* is dynamic and influenced by physiological factors such as stress, exercise and pathological conditions like hypertension and hypotension, isolated working‐heart models commonly present afterload as a static hydrostatic pressure. Although this method provides a controlled and simplified means of simulating afterload (Fig. 6*A*), it imposes an external pressure that the heart must overcome rather than allowing it to generate that pressure intrinsically through its own contraction, as it would *in vivo*. This simplification can be sufficient for experiments in which only steady‐state ventricular performance is of interest. However hydrostatic afterload becomes inadequate when ventricular function depends on vascular dynamic coupling or transient changes in loading conditions. An example includes experiments examining the beat‐to‐beat responses to arrhythmias or rapid pacing. In such settings the limitation arises because a hydrostatic column provides only a fixed outflow pressure and cannot change on the time scale of a single cardiac cycle. Beat‐to‐beat disturbances such as irregular cycle or rapid pacing may often be associated with immediate changes in systolic pressure, diastolic pressure, arterial compliance and peripheral resistance. These rapid fluctuations form part of the physiological afterload the ventricle experiences *in vivo*. A static column cannot adjust its height, compliance, resistance or wave‐reflection characteristics within milliseconds. It therefore cannot reproduce the dynamic afterload that accompanies arrhythmias or high‐frequency pacing.

Furthermore, physiological changes in afterload occur within each heartbeat; therefore experimental loading systems must be capable of adjusting pressure or flow impedance on a beat‐to‐beat basis, consistent with *in vivo* observations by Molino et al. (1998) who demonstrated that systemic arterial compliance and peripheral resistance change beat by beat. In contrast hydrostatic columns can be altered only manually or mechanically over seconds, which is too slow to replicate rapid physiological transients. Nonetheless prescribing afterload via a column height remains the standard in many experimental set‐ups and is employed in most commercially available isolated working‐heart systems.

## Afterload – variable pressure control

**Figure 7:**
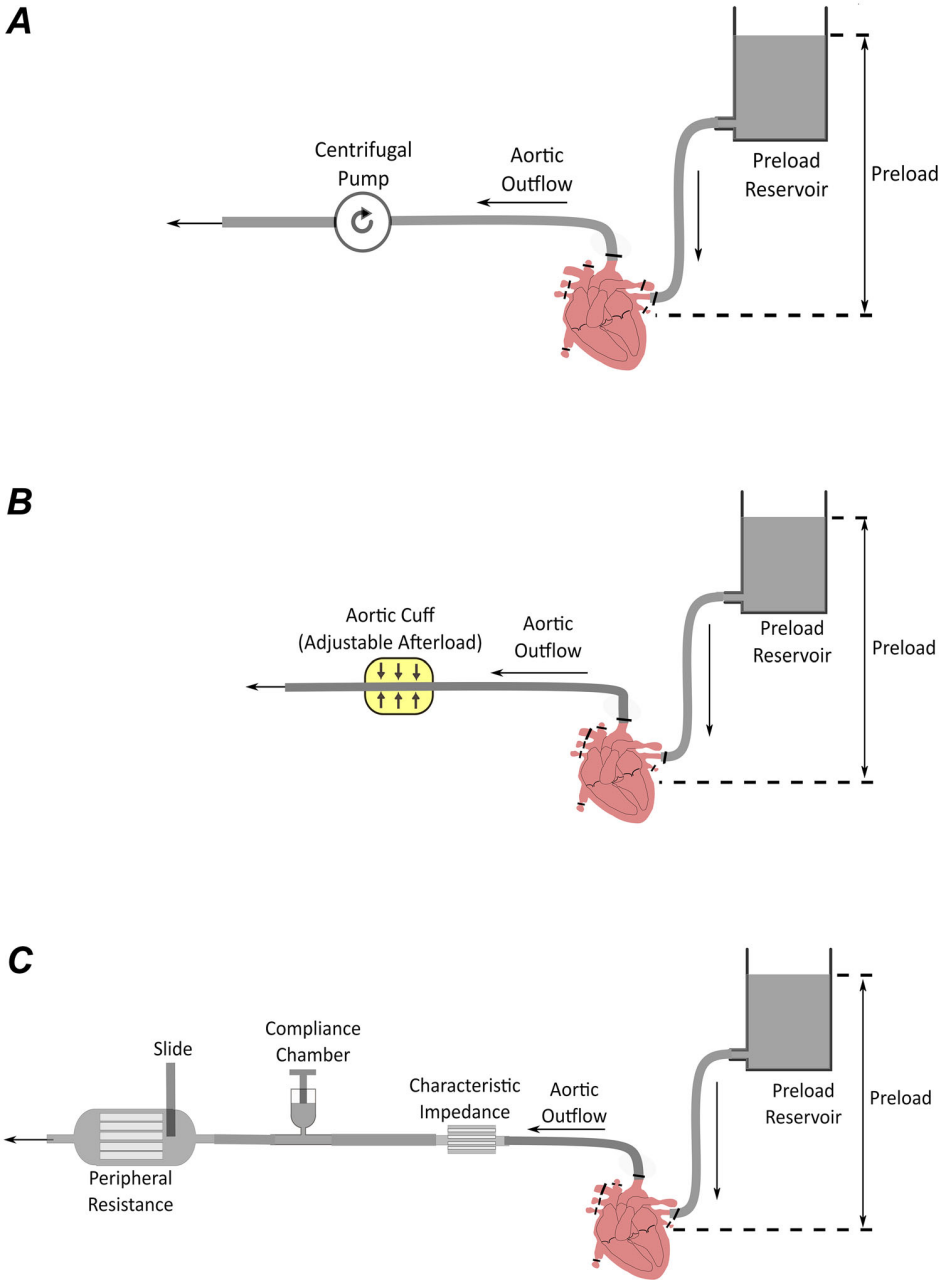
Afterload loading system *A*, Isolated working‐heart model that implements a variable pressure‐controlled afterload. The centrifugal pump rotates in the direction that opposes the flow out of the heart. The spinning centrifugal pump effectively modulates pressure by pushing blood back against the heart, thus creating a controlled and desired afterload. The pump functions as a controlled flow/pressure source rather than a representation of the arterial tree and, therefore, does not model vascular resistance, compliance or wave reflections. This illustration has been redrawn based on a figure originally published by Schechter et al. (Schechter et al., 2014). *B*, an impedance‐controlled afterload system employing a motor‐driven aortic cuff. The cuff acts as a variable resistor, modulating aortic outflow to achieve targeted left‐ventricular pressures. The left‐ventricular pressure is controlled by adjusting the resistance in the aortic column. The adjustable cuff represents peripheral vascular resistance, modelling the steady component of afterload without compliance. The drawing is adapted from a figure originally published by Pigot et al. (2022) (Pigot et al., 2022). *C*, physical three‐element Windkessel model for loading an isolated working heart. This drawing is adapted from figures published in the literature (Westerhof et al., 2009, 2010). The characteristic impedance element represents proximal aortic stiffness; the compliance chamber represents arterial compliance; and the peripheral resistance element represents systemic vascular resistance. Together, these components approximate the physiological arterial impedance experienced by the left ventricle.

To address some of the limitations of hydrostatic pressure‐controlled loading systems, alternative methods for afterload control have been developed. For instance, Schechter et al. (2014) designed a working‐heart system for large animals, such as pigs, in which afterload pressure was modulated by varying the speed of a centrifugal pump connected to the aortic line (Fig. 7*A*). The centrifugal pump replaces the conventional hydrostatic pressure column, providing a variable‐pressure afterload by generating an opposing pressure against the heart. This allows for active modulation of afterload by altering the pump's speed.

**Figure 8:**
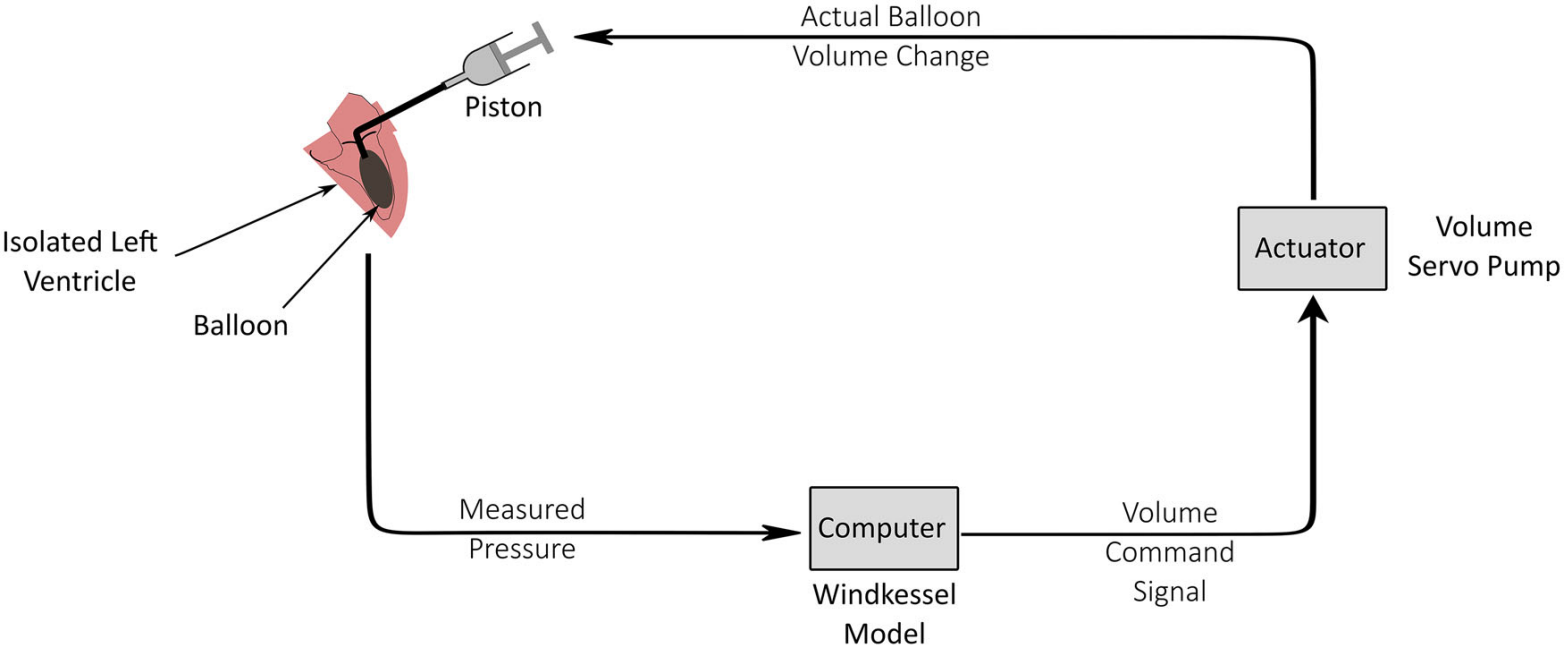
Impedance‐controlled afterload system based on a three‐element Windkessel model The physical Windkessel elements are modelled in software and adjusted programmatically. As indicated by the arrows, the Windkessel models take an input of measured left‐ventricular pressure and produce an output of a volume command signal that allows the volume servo pump to control the filling and ejection of perfusate in and out of the balloon via a piston connected to a linear motor. Schematic adapted from figure originally published by Sunagawa et al. (Sunagawa et al., 1982).

Despite the ability to vary the centrifugal pump's speed, the technique imposes a fixed afterload and, therefore, does not replicate the rapid, beat‐to‐beat fluctuations in afterload that occur *in vivo*. This limitation has motivated the development of impedance‐controlled afterload models, which aim to regulate the resistance to aortic outflow rather than directly imposing a pressure on the heart.

## Afterload – impedance control through a variable resistor

In impedance‐controlled systems, adjustable impedances are used to achieve desired afterloads. The concept dates back to Starling's heart–lung preparations in 1912 (Knowlton & Starling, 1912; Starling, 1930), where a variable resistor was placed on the aortic column to modulate aortic outflow resistance (Fig. 4). A modern example of this approach is the non‐linear afterload model developed by Pigot et al. (2022) that allows for independent control of systolic and diastolic aortic pressures using a motor‐driven cuff around the aortic line of an isolated pig working heart (Fig. 7*B*). In their set‐up, a soft silicone cuff is positioned around the aortic line and actuated by a plunger driven by a linear motor. The degree of constriction determines the resistance faced by the ejected perfusate and thus the afterload. Target systolic and diastolic pressures are set via a control interface, and a feedback loop continuously measures aortic pressure in real time, adjusting cuff constriction to maintain pressures within the desired range. This closed‐loop approach enables dynamic, non‐linear and physiologically targeted pressure control.

However, this system does not explicitly quantify the resistance or impedance faced by the heart. Additionally, the mechanical properties of the cuff introduce additional compliance and resistance that are not directly measured. The uncharacterised elements can contribute to variability in systolic pressure and unrepeatable pressure–flow relationships. As discussed in the next section, quantifying these embedded mechanical properties could improve the accuracy and reproducibility of afterload simulation.

In their more recent study, Pigot et al. (2023) developed a completely different afterload control. They demonstrated an active beat‐to‐beat afterload control concept, replacing the adjustable cuff with a plunger‐actuated valve. The plunger acts as an adjustable resistor, altering aortic outflow resistance in real time to achieve target left‐ventricular pressures and flows. Although this new approach offers faster and more precise control than fixed flow‐resistance systems (e.g. Frank's 1895 model (Frank, 1959)), the study is limited to simulations based on mathematical computations. That is, this new approach has not yet been applied to studying isolated hearts. When it does, care must be given to the physical implementation of the active beat‐to‐beat afterload control, as it could introduce additional flow dynamics, inertia or delays that may affect experimental performance.

Building on their 2022 work, Pigot et al. (2024), later in 2024, integrated the aortic cuff pressure‐feedback principle into a fully functional biventricular working‐heart model, incorporating a software‐controlled dynamic flow resistance. This allowed for more comprehensive assessment, including estimates of cardiac mechanical efficiency in isolated porcine working hearts. Nonetheless, the device retained the same limitations regarding unquantified impedance elements.

## Afterload – impedance control through Windkessel model

In addition to resistor‐based afterload systems described above, more advanced impedance‐controlled set‐ups incorporate resistor–capacitor networks to reproduce the haemodynamic load experienced by an isolated working heart. These systems, derived from the Windkessel model, extend simple resistive afterloads by including additional components that represent arterial compliance and characteristic impedance (Westerhof et al., 2009; Garrett et al., 2017).

In each heartbeat, the left ventricle contracts against a dynamic afterload (Garrett et al., 2017), characterised by the pressure–flow relationship of the arterial system. The Windkessel model, first proposed by Otto Frank in 1899, provides a lumped parameter representation of the pressure–flow relationship by linking the arterial compliance (the ability of the arterial wall to store volume for a given pressure change) with peripheral resistance (Frank, 1990; Sagawa et al., 1990; Kuhtz‐Buschbeck et al., 2018). The model captures the distensibility of the arterial system rather than its stiffness. As illustrated in Fig. 3, the model has become a cornerstone for simulating physiological afterload conditions in working‐heart experiments. Frank's Windkessel model, often referred to as the two‐element model, incorporates ‘arterial compliance (*C*)’ and ‘peripheral resistance (*R*_p_)’ to represent the storage and dissipation of blood within the arterial system. Compliance accounts for the ability of large arteries, such as the aorta, to expand and recoil with each heartbeat, thereby smoothing the pulsatile nature of ventricular ejection. Peripheral resistance refers to the resistance offered by the smaller arterioles and capillaries, which determines blood flow in the arterial system.

This framework was later extended to the three‐element Windkessel model, which introduces ‘characteristic impedance (*Z*_c_)’ to account for the wave‐propagation effects that occur in the proximal aorta (Westerhof et al., 1971; Garrett et al., 2017, 2019; Qureshi et al., 2018). The inclusion of *Z*_c_ enables a more accurate representation of the instantaneous pressure–flow relationship during systole and thereby improves the model's ability to replicate physiological arterial dynamics. The three‐element Windkessel model therefore provides a more comprehensive characterisation of ventricular afterload, as described in the following sections.

In the three‐element Windkessel model, blood flow is analogous to electrical current and left‐ventricular pressure to voltage. Peripheral resistance is represented by a resistor; aortic compliance is modelled by a capacitor; and the aorta's characteristic impedance is denoted by an additional resistor in series with the voltage source (Garrett et al., 2019). This configuration captures both steady and pulsatile aspects of afterload. The electrical circuit equivalence of this model has been shown in Fig. 6*B*.

Windkessel elements can be implemented in physical hydrodynamic models and can provide an adjustable representation of afterload (Fig. 7*C*). The characteristic impedance is modelled by a tube with conduits that impede flow from the heart. Aortic compliance is simulated by an air‐filled chamber with a movable plunger to adjust the volume of air inside and, thus, the capacitance. Peripheral resistance is modelled by a network of narrow tubes, with flow regulated by a sliding gate. Such set‐ups generate ventricular and aortic pressure–flow waveforms that closely resemble those observed *in vivo*. Representative examples of these haemodynamic waveforms have been reported in multiple studies (Molino et al., 1998; Gerringer et al., 2018; Moravia, 2021; Miranda‐Silva et al., 2022). Owing to their ability to vary resistance, compliance and impedance, physical Windkessel models can be used in valve testing, artificial heart evaluation and disease modelling (Westerhof et al., 2009).

One notable early example is the three‐element Windkessel constructed by Elzinga and Westerhof (1971) to mimic the arterial system. The model is similar to the one illustrated in Fig. 7*C*. Their design provided experimentally determined values for compliance, characteristic impedance and peripheral resistance for both the systemic and pulmonary circulations in humans, dogs, cats and rats. They also detailed practical implementation parameters such as the air volumes required to achieve desired capacitances and the tubing dimensions needed to achieve specific resistance and impedance values. Additionally *in vivo* values for the Windkessel parameters were also determined experimentally in rats (Molino et al., 1998).

Building on their 1971 characterisation work, Elzinga and Westerhof (1973) applied their hydraulic Windkessel model to load an isolated cat heart, enabling controlled investigation of how selective changes in peripheral resistance and arterial compliance affect left‐ventricular pressure and aortic outflow. The characteristic impedance was kept constant. The results showed that increasing peripheral resistance elevated both left‐ventricular and aortic pressures, whereas reducing total compliance (aortic compliance) increased systolic pressures and lowered diastolic pressures.

Although this 1973 system presented a significant advance by implementing afterload as an impedance rather than static hydrostatic pressure or variable resistance, it lacked real‐time dynamic control. Once resistance and compliance were set, they remained fixed throughout the experiment, with no feedback mechanism to adjust loading in response to instantaneous changes in pressure or flow. The absence of adaptive feedback limited its ability to replicate the beat‐to‐beat variability of afterload observed *in vivo*.

Building on this foundation Ishide et al. (1980) adapted the three‐element Windkessel system of Westerhorff et al. (1971) for their investigation of the external loading of the left ventricle in isolated dog hearts. Their aim was to examine how altering aortic impedance influenced left‐ventricular pressure and volume during systole. However, as with Elzinga and Westerhof (1973) model, the absence of closed‐loop feedback meant that the afterload parameters could not adapt in real time to beat‐to‐beat changes in ventricular output.

Extending the previous Windkessel‐based concepts described above, Sunagawa et al. (1982) introduced a servo‐controlled system to actively regulate the impedance imposed on an isolated left ventricle, not an isolated whole heart. Unlike the earlier hydraulic models with fixed resistance and compliance, this system employed an analogue computer to implement a three‐element Windkessel model, prescribing a programmable arterial impedance and computing instantaneous aortic flow and left‐ventricular volume. The innovation in this system is that it removes the physical elements of the Windkessel model, as illustrated in Fig. 8. The system consisted of three main components: (1) a volume servo pump that controlled ventricular volume based on a command signal from the loading system, (2) a loading system that computed ejection and filling flows for each cycle and (3) a control system, implemented via a digital computer, that allowed dynamic adjustment of afterload and preload parameters. A balloon catheter inserted into the isolated left ventricle simulated ejection and filling via active inflation and deflation, with the servo pump connected to the balloon through a piston driven by a linear motor. This system enabled precise volume control, allowing the ventricular volume to be tracked accurately throughout systole and diastole.

Unlike the flow‐through models described above, this system of Sunagawa et al. (1982) did not circulate the perfusate. Instead it operated in a closed, non‐circulatory loop where the same volume of fluid was alternately ejected and reinjected into the balloon in each cardiac cycle. Although the system allowed programmable variation in afterload parameters, real‐time impedance control and mimicked physiological pressure–volume relations with high fidelity, it lacked a true flow‐through perfusion circuit. Thus, the major limitation of this system is that it represents a partially functioning left ventricle, with no systemic circulation, rather than a complete working‐heart preparation.

An extension of Sunagawa et al.’s (1982) servo‐controlled impedance model was achieved by Maughan et al. (1984) who integrated both preload and afterload simulation to investigate the effects of arterial impedance on the end‐systolic pressure–volume relationship. The servo‐controlled pump system was connected to an excised dog ventricle, and the simulated Windkessel parameters were applied. Despite being a partial working‐heart model without flow‐through perfusion, this approach established a framework for subsequent studies (Sunagawa et al., 1983; Alexander et al., 1987; De Tombe et al., 1993) employing servo‐controlled, Windkessel‐based afterload simulations.

## Perspective

This review provides a comprehensive account of the methods used to present preload and afterload to isolated‐heart preparations, particularly in working‐heart models. Preload is typically adjusted via balloon inflation in Langendorff preparations; however in working‐heart preparations, it is typically regulated by adjusting the height of the preload reservoir. Afterload has been presented in various forms, including static hydrostatic pressure, variable pressure via pumps and impedance using mechanical or software‐based systems. The latter includes resistor‐based models and more advanced Windkessel models that simulate arterial compliance, characteristic impedance and peripheral resistance. Despite these advances, many systems still lack real‐time feedback control of afterload, which limits their ability to replicate the beat‐to‐beat variability observed *in vivo*. This limitation is not merely technical; it represents a fundamental physiological gap between *ex vivo* models and the dynamic arterial environment of the intact circulation.

From our review of current loading methods, we provide three perspectives. First, there remains a need for a system capable of functioning as a real‐time variable‐flow resistor, offering a new approach beyond the traditional adjustable cuff, which lacks dynamic responsiveness and inherently constrains physiological fidelity. With such a real‐time system, the aortic flow could be programmatically controlled and modulated in real time to dynamically change impedance and, thereby, afterload. This new system would take real‐time measurement of left‐ventricular pressure as input and compute the desired aortic outflow and pressure as outputs. The system could ideally adjust the aortic tubing constriction to match the predicted aortic flow with the measured flow in real time. In our view, without this level of responsiveness, claims of physiologically realistic afterload simulation remain unrealised.

Second, coronary perfusion must be considered in the design, as it plays a critical role in relieving the pressure distal to the aortic valve. Current afterload–control methods do not explicitly account for coronary flow, which fundamentally distorts the pressure–flow environment experienced by the left ventricle. Ignoring this pathway risks introducing systematic error in both flow and pressure regulation. This flow can be as high as the aortic flow, particularly at high afterloads (Goo et al., 2014a; Han et al., 2018), as in the case where the aortic line is clamped, in which all of the inflow is ejected to the coronary ostia. Accounting for coronary flow is essential to ensure that the flow control mechanism can reliably and accurately match the measured aortic flow with the system‐predicted flow. We propose that future systems incorporate coronary perfusion into their control algorithms if they are to reproduce physiologically realistic systolic pressure development and diastolic pressure decay.

Lastly, a feedback‐controlled flow impedance could be employed to ensure that the measured aortic pressure matches the predicted value, thereby restoring the natural function of the aortic valve as a dynamic regulator of ventricular ejection. More importantly, it would shift isolated heart experimentation from static load imposition towards true closed‐loop ventricular–vascular coupling (VVC). Without closed‐loop control, afterload remains fundamentally static, regardless of how it is presented. Such systems would allow the beat‐to‐beat modulation of afterload, expanding the experimental capabilities of isolated working‐heart research. Quantifying ventricular–vascular coupling under these conditions is particularly valuable because it provides an integrated measure of how effectively the ventricle converts mechanical energy into forward flow for a given arterial load. As highlighted by Antonini‐Canterin et al. (2013), the arterial elastance (*E*_a_) and the end‐systolic elastance (*E*_es_), *E*_a_/*E*_es_, capture the balance between ventricular systolic performance and arterial elastance, with optimal coupling (*E*_a_/*E*_es_ ≈ 1) maximising ventricular pressure–volume stroke work and minimising energetic cost. Therefore, peak work and efficiency occur when VVC = 1 (De Tombe et al., 1993). In contrast, mismatched coupling, whether due to increased arterial stiffness or impaired contractility, produces mechanical inefficiency and elevated myocardial oxygen demand. More recent perspectives reinforce this mechanistic importance. Monge Garcia and Santos (2020) highlight that VVC integrates ventricular contractility, arterial load and myocardial energetics, and that disruptions in this interaction (whether from ageing, hypertension or critical illness) directly impair ventricular efficiency, reinforcing that VVC is a physiologically grounded descriptor of how the heart and arterial system function as an integrated pump. Toktarbay et al. (2024) emphasise that VVC uniquely incorporates both steady and pulsatile components of afterload, including arterial compliance and characteristic impedance, making it a more physiologically complete descriptor of afterload than pressure alone. Crucially, population‐level evidence from Lin et al. (2026) demonstrates that non‐invasive VVC parameters independently predict arterial fibrillation, stroke, heart failure, coronary disease and mortality, and outperform traditional imaging markers such as ejection fraction. Together, these studies show that VVC is not merely a theoretical construct but a sensitive marker for early pump dysfunctions, a mechanistic link between arterial pathology and ventricular energetics and a clinically meaningful parameter that reveals disease processes invisible to conventional measures. For isolated‐heart research, where afterload is experimentally prescribed, quantifying VVC is important for determining whether imposed loading conditions re‐create physiological ventricular–vascular interaction and for understanding how pathological afterloads alter myocardial performance, efficiency and energetics. We contend that integrating real‐time flow control, coronary flow–aware modelling and pressure feedback is not simply an incremental refinement but a necessary progression if the working‐heart systems are to reproduce the dynamic complexity of cardiovascular physiology.

Beyond these technical considerations, advancing afterload–control systems are essential because several fundamental physiological questions remain inaccessible with current isolated‐heart techniques. Classical Langendorff perfusion cannot reproduce the mechanical work of ejection and therefore cannot address how pulsatile aortic pressure–flow modulates myocardial energetics or beat‐to‐beat ventricular–vascular interaction. Traditional working‐heart systems, while enabling stroke volume and cardiac output measurements, still lack the dynamic impedance characteristics needed for loading the heart to study how the ventricle responds and adapts to rapid changes in arterial stiffness or pathological afterloads. Thus, in the current set‐up the understanding of vascular–ventricle coupling in a dynamic sense is lacking. Without physiologically realistic afterload, it is not possible to determine how abnormal loading initiates early hypertrophic signalling that alters coronary flow. Next‐generation impedance‐controlled systems therefore have the potential to unlock new mechanistic insights into load‐induced disease, myocardial energetics and the integrated behaviour of the heart under realistic arterial conditions, questions that cannot be answered with existing methods.

## Additional information

### Competing interests

All authors declare no conflicts of interest, financial or otherwise.

### Author contributions

E.M. and J.‐C.H. drafted the manuscript. A.T., T.P. and K.T. provided manuscript revisions, editing and proofreading. All authors read and approved the final manuscript.

### Funding

This work was supported by funding received from the Heart Foundation of New Zealand through Project Grant (1929 to J.‐C.H.) and Senior Research Fellowship Grant (2027 to J.‐C.H.); the Royal Society of New Zealand through Marsden Project Grant (MFP‐UOA2206 to J.‐C.H.); and the Health Research Council of New Zealand through Sir Charles Hercus Health Research Fellowship Grants (21/116 to K.T.) and Emerging Researcher First Grant (21/653 to T.P.).

### Generative AI statement

The authors confirm that no artificial intelligence tools, including large language models (LLMs), were used in the drafting or revision of this manuscript. All content was conceived, written and approved solely by the authors.

## Supporting information


Peer Review History
