# Supplementary material for: Presentation of preload and afterload to isolated heart preparations
Source: J Physiol. 2026 Jul 31;604(17):7025–44. doi: 10.1113/JP291198 (PMC13532823; doi:10.1113/JP291198)
Supplement: Supplementary file 1 [file TJP-604-7025-s001.pdf]

The Journal of Physiology

Presentation of preload and afterload to isolated heart preparations

Esau Mhandu, Toan Pham, Kenneth Tran, Andrew Taberner, and June-Chiew Han
DOI: 10.1113/JP291198

Corresponding author(s): June-Chiew Han (j.han@auckland.ac.nz)

The following individual(s) involved in review of this submission have agreed to reveal their identity: Klaus-Dieter Schlüter (Referee #2)

Review Timeline:

Submission Date:	08-Mar-2026
Editorial Decision:	13-Apr-2026
Revision Received:	11-Jun-2026
Accepted:	01-Jul-2026

Senior Editor: Laura Bennet

Reviewing Editor: Wolfgang Linke

Transaction Report:

(Note: With the exception of the correction of typographical or spelling errors that could be a source of ambiguity, letters and reports are not edited. Depending on transfer agreements, referee reports obtained elsewhere may or may not be included in this compilation. Referee reports are anonymous unless the Referee chooses to sign their reports.)

Re: JP-TR-2026-291198 "**Presentation of preload and afterload to isolated heart preparations**" by Esau Mhandu, Toan Pham, Kenneth Tran, Andrew Taberner, and June-Chiew Han

Dear Dr Han,

Thank you for submitting your manuscript to The Journal of Physiology. It has been assessed by a Reviewing Editor and by 2 expert referee(s) and we are pleased to tell you that it is potentially acceptable for publication following satisfactory major revision.

The referee reports are copied at the end of this email.
Please address all the points raised and incorporate all requested revisions or explain in your Response to Referees why a change has not been made. We hope you will find the comments helpful and that you will be able to return your revised manuscript within 2 months. If your article is NOT for a Special Issue, you may have 9 months to revise. If you require an extension, please contact journal staff: jp@physoc.org. Please note that this letter does not constitute a guarantee for acceptance of your revised manuscript.

ABSTRACT FIGURES: Authors are expected to use The Journal's premium BioRender account to create/redraw their Abstract Figures. Information on how to access this account is here:
https://physoc.onlinelibrary.wiley.com/journal/14697793/biorender-access.
This will enable Authors to create and download high-resolution figures. If authors have used the free BioRender service, they can use the instructions provided in the link above to download a high-resolution version suitable for publication.
The link provided should only be used for the purposes of this submission. Authors will be charged for figures created on this account if they are not related to this manuscript submission.

Your revised manuscript should be submitted online using the link in your Author Tasks: Link Not Available. This link is accessible via your account as Corresponding Author; it is not available to your co-authors. If this presents a problem, please contact journal staff (jp@physoc.org). Image files from the previous version are retained on the system. Please ensure you replace or remove any files that are being revised.
If you do not wish to submit a revised version of your manuscript, you must inform our journal staff (jp@physoc.org) or reply to this email to request withdrawal. Please note that a manuscript must be formally withdrawn from the peer review process at one journal before it may be submitted to another journal.

REVISION CHECKLIST:
Check that your Methods section conforms to journal policy: https://jp.msubmit.net/cgi-bin/main.plex?form_type=display_requirements#methods.

Check that data presented conforms to the statistics policy: https://jp.msubmit.net/cgi-bin/main.plex?form_type=display_requirements#statistics.

Upload a full Response to Referees file. To create your 'Response to Referees': copy all the reports, including any comments from the Senior and Reviewing Editors, into a Microsoft Word, or similar, file and respond to each point, using font or background colour to distinguish comments and responses and upload as the required file type.

Please upload two versions of your manuscript text: one with all relevant changes highlighted and one clean version with no changes tracked. The manuscript file should include all tables and figure legends, but each figure/graph should be uploaded as separate, high-resolution files.

You may also upload:
- 'Potential Cover Art' for consideration as the issue's cover image
- Appropriate Supporting Information (Video, audio or data set: see https://jp.msubmit.net/cgi-bin/main.plex?form_type=display_requirements#supp).

IMPORTANT POINTS TO NOTE WHEN REVISING YOUR MANUSCRIPT:
LANGUAGE EDITING AND SUPPORT FOR PUBLICATION: If you would like help with English language editing, or other article preparation support, Wiley Editing Services offers expert help, including English Language Editing, as well as translation, manuscript formatting, and figure formatting at www.wileyauthors.com/eeo/preparation. You can also find resources for Preparing Your Article for general guidance about writing and preparing your manuscript at www.wileyauthors.com/eeo/prepresources.

TRANSPARENT PEER REVIEW POLICY: To improve the transparency of its peer review process, The Journal of Physiology publishes online as supporting information the peer review history of all articles accepted for publication. Readers will have access to decision letters, including Editors' comments and referee reports, for each version of the manuscript, as well as any author responses to peer review comments. Referees can decide whether or not they wish to be named on the peer review history document.

We look forward to receiving your revised submission.

If you have any queries, please reply to this email and we will be pleased to advise.

Yours sincerely,

Laura Bennet
Senior Editor
The Journal of Physiology

REQUIRED ITEMS FOR REVISION

- Please include an Abstract Figure file and an Abstract Figure legend. An appropriate figure legend, which should not exceed 150 words in length, should be included in the main manuscript file. The Abstract Figure is a piece of artwork designed to give readers an immediate understanding of the research and should summarise the main conclusions. If possible, the image should be easily 'readable' from left to right or top to bottom. It should show the physiological relevance of the manuscript so readers can assess the importance and content of its findings. Abstract Figures should not merely recapitulate other figures in the manuscript. Please try to keep the diagram as simple as possible and without superfluous information that may distract from the main conclusion(s). Abstract Figures must be provided by authors no later than the revised manuscript stage and should be uploaded as a separate file during online submission labelled as File Type 'Abstract Figure'. Please also ensure that you include the figure legend in the main article file. All Abstract Figures should be created using BioRender. Authors should use The Journal's premium BioRender account to export high-resolution images. Details on how to use and access the premium account are included as part of this email.

- Your MS must include a complete "Additional information section" with the following 4 headings and content:

Competing Interests: A statement regarding competing interests. If there are no competing interests, a statement to this effect must be included. All authors should disclose any conflict of interest in accordance with journal policy.

Author contributions: Each author should take responsibility for a particular section of the study and have contributed to writing the paper. Acquisition of funding, administrative support or the collection of data alone does not justify authorship; these contributions to the study should be listed in the Acknowledgements. Additional information such as 'X and Y have contributed equally to this work' may be added as a footnote on the title page.

It must be stated that all authors approved the final version of the manuscript and that all persons designated as authors qualify for authorship, and all those who qualify for authorship are listed.

Funding: Authors must indicate all sources of funding, including grant numbers. If authors have not received funding, this must be stated.

It is the responsibility of authors funded by RCUK to adhere to their policy regarding funding sources and underlying research material. The policy requires funding information to be included within the acknowledgement section of a paper. Guidance on how to acknowledge funding information is provided by the Research Information Network. The policy also requires all research papers, if applicable, to include a statement on how any underlying research materials, such as data, samples or models, can be accessed. However, the policy does not require that the data must be made open. If there are considered to be good or compelling reasons to protect access to the data, for example commercial confidentiality or legitimate sensitivities around data derived from potentially identifiable human participants, these should be included in the statement.

Acknowledgements: Acknowledgements should be the minimum consistent with courtesy. The wording of acknowledgements of scientific assistance or advice must have been seen and approved by the persons concerned. This section should not include details of funding.

EDITOR COMMENTS

Reviewing Editor:

The reviewers agreed that this manuscript has the potential to serve as a useful and engaging overview. However, they also provided several specific suggestions for improvement. Please address these comments comprehensively, with particular attention to more clearly conveying the article's significance for the field and strengthening its overall narrative coherence.

REFEREE COMMENTS

Referee #1:

This is a thorough review of load presentation in isolated working heart preparations. The review provides historical progression and is nicely organized by preload and afterload, which is sub-categorized by hydrostatic pressure, variable pressure control, and impedance control through a variable resistor multi-variable. The authors appropriately provide future perspectives for improved ways to mimic in vivo conditions more accurately.

The review could be improved by a more straightforward design, which defines the experimental objective throughout the review. Are experimental aims designed to measure afterload (i.e., as the dependent variable) or are the aims to simulate physiological afterload (i.e., it serves as the independent variable)? If the latter, what are the key questions (dependent variables) in the field (e.g., how afterload modulates oxygen consumption, coronary flow, metabolism, protein synthesis, ventricular performance (contractility, pressure, stroke volume, cardiac output, power)? Additionally, is afterload control necessary to advance mechanistic studies, disease modeling, tissue/organ engineering, and/or drug/small molecule testing?

A running theme of approach, limitations, advancement, remaining gaps, and next key questions would add value to the review and provide a robust assessment for high impact experiments for next generation cardiovascular scientists.

Minor

Page 6, Lines 119-121. Is impedance equivalent to physiological afterload? If so, this should be a running theme about the objective to mimic in isolated heart preparations.

Page 8, Lines 199-202. What is meant by fibre length? Can this be quantified?

Page 19, Lines 468-471. The limitations are not intuitive for examining beat-to-beat responses to arrhythmias or rapid pacing with hydrostatic afterload. Please elaborate.

Page 20, Figure 7. Please consider adding physiological analogs to the applied devices in panels A, B, & C.

Page 22, line 558. Elasticity is the inverse of compliance.

Page 23, Lines 587-588. Please consider adding a representative ventricular and aortic pressure-flow waveforms that resemble those in vivo.

Page 28, Lines 699-701. Please consider elaborating on the value of quantifying ventricular-vascular coupling as a parameter to study cardiac pump function in health and disease.

Referee #2:

In the current and very interesting review article that authors nicely explain the differences between classical Langendorff techniques and Working heart preparation. The authors nicely work out the current limitations as summarized at the end of the review. However, what is missing is a discussion about what kind of new information we will get from such an improvement and what kind of questions can be addressed using either the traditional Langendorf or the more sophisticated working heart mode. In the introduction the authors claim that this would be important for energy consumption. This is right but still perfusion solutions are artificial and will have a major impact on energy turnover. So, the authors should give the reader examples which questions in heart physiology can NOT be addressed with the current techniques and require the progression of technical development as summarized at the end of the review. With these extensions the review would be much better and much more interesting. As it stands it is a beautiful historical overview with an outlook to future improvement but without making the point of relevance for heart physiology.

Specific comments:

Line 79/80: Thus, an isolated heart preparation is devoid of systemic circulation and neuromodulation(Grover & Singh, 2007; Watanabe & Okada, 2018).

The sentence is the logic consequence of isolation of the heart. What should the citations add to this simple sentence?

Line 94-96: The isolated heart preparation was first demonstrated by Carl Ludwig and Elias Cyon in 1866 using the frog heart(Zimmer, 1998).

Why not give the original citation? If you cite the elegant text from Zimmer, then this should be claimed such as 'as reminded by Zimmer, 1998)'.

Line 150-153: An alternative technique that does not require a donor animal is the replacement of blood with washed red blood cells to perfuse the isolated heart.

This is not good explained. Blood consists of plasma and hematocrit. You can hardly perfuse a heart with only washed red blood cells (hematocrit). How is the plasma part covered in these examples? The authors should add examples in which erythrocytes were replaced by artificial oxygen transport carriers (i.e. PMID 28278587).

Line 169: Isolated Perfusion Heart Models Across Species.

In this chapter I miss a critical discussion about how the aforementioned types of saline-based perfusate differ between species and about their artificial side effects such as high glucose instead of glucose / fatty acids, low perfusion pressure due to the lack of colloid osmotic pressure from albumin and higher shear stress. Please add the importance and influence of pyruvate often added as well.

As the review compared isovolumetric Langendorff technique with the ore complex working heart model and suggests that the latter one reflects the physiological situation much better than the former, the authors should add examples in which Langendorff technique resulted in false interpretation of the results that was later than corrected by the working heart model. This is important to include as the authors make the point the working mode should preferentially be used.

Line 303: Under these conditions, the heart continued to beat for up to five hours.

This is indeed important for many experimental setups. Please give more information about the stability of isolated heart preparations in the Langendorff mode and the working heart mode.

Lime 315: Based on experiments with isolated frog 315 hearts, he developed mathematical models to investigate how changes in vascular elasticity 316 and resistance shaped the arterial pulse(Frank, 1990...).

I am afraid that Otto Frank is not alive in 1990! He can hardly developed such models in 1990!

END OF COMMENTS

EDITOR COMMENTS

Reviewing Editor:

The reviewers agreed that this manuscript has the potential to serve as a useful and engaging overview. However, they also provided several specific suggestions for improvement. Please address these comments comprehensively, with particular attention to more clearly conveying the article's significance for the field and strengthening its overall narrative coherence.

Thank you for the opportunity to improve our manuscript. We have carefully addressed all comments from both reviewers and revised the manuscript accordingly.

In response to Reviewer 1, we have clarified the experimental objectives, explicitly defined afterload as an independent variable, and strengthened the conceptual framework by emphasising key dependent measures and the role of controlled afterload across applications. We have also improved the manuscript's structure and coherence by incorporating a clearer thematic progression and elaborating on methodological limitations and future directions in the Perspective section.

In response to Reviewer 2, we have expanded the discussion of physiological relevance by outlining the specific scientific questions that cannot be addressed with current techniques, and by highlighting the new insights enabled by advanced afterload control systems.

REVIEWER #1

Major Comments

This is a thorough review of load presentation in isolated working heart preparations. The review provides historical progression and is nicely organized by preload and afterload, which is sub-categorized by hydrostatic pressure, variable pressure control, and impedance control through a variable resistor multi-variable. The authors appropriately provide future perspectives for improved ways to mimic in vivo conditions more accurately.

The review could be improved by a more straightforward design, which defines the experimental objective throughout the review. Are experimental aims designed to measure afterload (i.e., as the dependent variable) or are the aims to simulate physiological afterload (i.e., it serves as the independent variable)? If the latter, what are the key questions (dependent variables) in the field (e.g., how afterload modulates oxygen consumption, coronary flow, metabolism, protein synthesis, ventricular performance (contractility, pressure, stroke volume, cardiac output, power)? Additionally, is afterload control necessary to advance mechanistic studies, disease modeling, tissue/organ engineering, and/or drug/small molecule testing?

A running theme of approach, limitations, advancement, remaining gaps, and next key questions would add value to the review and provide a robust assessment for high impact experiments for next generation cardiovascular scientists.

Thank you for this insightful comment. We have revised the Introduction to clearly define the experimental objective of the review (lines 145 to 157). Specifically, we now state that the purpose of this review is to present how physiological afterload can be prescribed in isolated-heart experiments. To make this explicit, we clarify that afterload is treated as an independent variable, and we identify the key dependent variables commonly investigated under controlled conditions (e.g., ventricular and aortic pressure generation, stroke volume, cardiac output, oxygen consumption and coronary flow).

We have also expanded the Introduction to explain why controlled afterload is essential for mechanistic studies, disease modelling, tissue and organ engineering, and load-dependent pharmacological testing (lines 141 - 146).

These additions provide a clearer conceptual framework and ensure that the experimental aims are consistently defined throughout the review.

Minor Comments

Page 6, Lines 119-121. Is impedance equivalent to physiological afterload? If so, this should be a running theme about the objective to mimic in isolated heart preparations.

In the intact circulation, arterial impedance is considered the most complete descriptor of ventricular afterload, because it incorporates both the steady (resistive) and pulsatile (compliant and inertial) components of the arterial systems. However, when studying isolated working-heart preparations, researchers often employ simplified strategies such as hydrostatic pressure or resistance that capture only selected aspects of afterload. In the revised manuscript, we clarify that impedance is not used as a synonym for afterload but rather represents the physiologically relevant definition that the various experimental loading methods attempt to approximate to differing degrees. We have revised the Introduction (lines 122 to 132) to make this relationship explicit and to emphasise that a central theme of the review is how different loading techniques reproduce, simplify or omit components of physiological afterload.

Page 8, Lines 199-202. What is meant by fibre length? Can this be quantified?

In the revised manuscript, we clarify that 'fibre length' refers to the end-diastolic sarcomere length or muscle length of myocardial fibres, which can be quantified directly in isolated muscle preparations (e.g. via laser diffraction for sarcomere length measurement or microscopic graticule for muscle length measurement) and in whole-heart preparations it is typically inferred from end-diastolic volume. We have added a sentence to make this explicit (lines 239 to 241).

Page 19, Lines 468-471. The limitations are not intuitive for examining beat-to-beat responses to arrhythmias or rapid pacing with hydrostatic afterload. Please elaborate.

We have now elaborated that hydrostatic column provides only a fixed pressure head and cannot adjust resistance, compliance or wave reflections on a cycle-to-cycle basic. Because arrythmias and rapid pacing produce beat-to-beat changes in arterial load, a static column cannot reproduce these rapid transients. We have revised the text accordingly to make this limitation explicit (lines 520 to 528).

Page 20, Figure 7. Please consider adding physiological analogs to the applied devices in panels A, B, & C.

We have revised Figure 7 legend to explain what each component represents. However, for the Panel A and Panel B, they have no direction physiological analogs. For example, there is nothing in the body that represents a centrifugal pump. Both panels are methods for modulating the aortic outflow rate. However, Panel C now labels the characteristic impedance, compliance chamber, and peripheral resistance as analogs of proximal aortic stiffness, arterial compliance and systemic vascular resistance, respectively. Together, these form a physical three-element Windkessel model of physiological afterload.

Page 22, line 558. Elasticity is the inverse of compliance

We agree that elasticity is the inverse of compliance. In the revised manuscript, we now refer specifically to arterial compliance when describing the Windkessel model and note that compliance represents the distensibility of the arterial wall (the inverse of elasticity) (lines 616 to 621).

Page 23, Lines 587-588. Please consider adding a representative ventricular and aortic pressure-flow waveforms that resemble those in vivo.

Thank you for the helpful comment. We have cited studies where these representative pressure-flow waveforms are shown (lines 648 to 650).

Page 28, Lines 699-701. Please consider elaborating on the value of quantifying ventricular-vascular coupling as a parameter to study cardiac pump function in health and disease.

Thank you for this insightful comment. We have expanded the paragraph to elaborate on the value of quantifying ventricular-vascular coupling (see lines 757 to 789). In the revised text, we emphasise that coupling describes how effectively the ventricle and arterial system operate together, reflecting the balance between ventricular contractile performance and the arterial system's resistance, compliance and pulsatile characteristics. Because this interaction becomes impaired in conditions such as hypertension, heart failure and arterial stiffening, coupling metrics provide a powerful means of assessing cardiac function in both healthy and diseased hearts. This addition clarifies the physiological importance of closed-loop afterload control in isolated working-heart experiments.

REVIEWER 2

Major Comments

In the current and very interesting review article that authors nicely explain the differences between classical Langendorff techniques and Working heart preparation. The authors nicely work out the current limitations as summarized at the end of the review. However, what is missing is a discussion about what kind of new information we will get from such an improvement and what kind of questions can be addressed using either the traditional Langendorf or the more sophisticated working heart mode. In the introduction the authors claim that this would be important for energy consumption. This is right but still perfusion solutions are artificial and will have a major impact on energy turnover. So, the authors should give the reader examples which questions in heart physiology can NOT be addressed with the current techniques and require the progression of technical development as summarized at the end of the review. With these extensions the review would be much better and much more interesting. As it stands it is a beautiful historical overview with an outlook to future improvement but without making the point of relevance for heart physiology.

Thank you for this valuable comment. We agree that the review would benefit from a clearer discussion of the physiological questions that cannot be addressed with current Langendorff or traditional working-heart techniques, and what new information improved afterload-control systems will enable. In response, we have added a new paragraph to the Perspective section that explicitly outlines the physiological limitations of existing methods, the key questions in myocardial energetics, ventricular-vascular coupling, coronary-myocardial interaction, and load-induced pathophysiology that remain inaccessible, and new mechanistic insights that next-generation impedance-controlled systems could provide (see lines 793 to 807). These additions improve the physiological relevance of the review and clarify why advancing afterload-control technology is essential for future high-impact cardiovascular research.

Minor Comments

Line 79/80: Thus, an isolated heart preparation is devoid of systemic circulation and neuromodulation (Grover & Singh, 2007; Watanabe & Okada, 2018).

The sentence is the logic consequence of isolation of the heart. What should the citations add to this simple sentence?

We agree that the absence of systemic circulation and neuromodulation follows directly from the definition of an isolated heart preparation. We have therefore removed the citations from this sentence but re-placed them to the preceding sentence (line 77).

Line 94-96: The isolated heart preparation was first demonstrated by Carl Ludwig and Elias Cyon in 1866 using the frog heart(Zimmer, 1998).
Why not give the original citation? If you cite the elegant text from Zimmer, then this should be claimed such as 'as reminded by Zimmer, 1998)'.
We agree Zimmer(1998) without clarification implies it is the original source. We have revised the sentence to add the original source and state that this historical information is as described by Zimmer(1998), which accurately reflects the secondary nature of the citation (lines 92 to 93).

Line 150-153: An alternative technique that does not require a donor animal is the replacement of blood with washed red blood cells to perfuse the isolated heart.
This is not good explained. Blood consists of plasma and hematocrit. You can hardly perfuse a heart with only washed red blood cells (hematocrit). How is the plasma part covered in these examples? The authors should add examples in which erythrocytes were replaced by artificial oxygen transport carriers (i.e. PMID 28278587).
We agree that the previous wording was unclear. Washed red blood cells are not used alone; they are suspended in a physiological buffer to replace plasma phase and create an oxygen-carrying perfusate. We have clarified this in the revised text (lines 172 to 186 and lines 180 to 183). In addition, as suggested, we now include examples of perfusion systems that replace erythrocytes entirely with oxygen carriers such as hemoglobin-based oxygen transport solutions.

Line 169: Isolated Perfusion Heart Models Across Species.
In this chapter I miss a critical discussion about how the aforementioned types of saline-based perfusate differ between species and about their artificial side effects such as high glucose instead of glucose / fatty acids, low perfusion pressure due to the lack of colloid osmotic pressure from albumin and higher shear stress. Please add the importance and influence of pyruvate often added as well.

We thank the reviewer for this valuable comment. We have now added a dedicated paragraph discussing the species-dependent difference in saline-based perfusates. This includes the use of supraphysiological glucose in rodents and the limited inclusion of fatty acids. We also discussed the absence of albumin and resulting loss of colloid osmotic pressure leading to myocardial edema and reduce peripheral perfusion. We have also incorporated a discussion on the importance of pyruvate supplementation and its beneficial effects on mitochondrial redox balance, oxidative ATP production and contractile recovery. (lines 216 to 234)

As the review compared isovolumetric Langendorff technique with the more complex working heart model and suggests that the latter one reflects the physiological situation much better than the former, the authors should add examples in which Langendorff technique resulted in false interpretation of the results that was later than corrected by the working heart model. This is important to include as the authors make the point the working mode should preferentially be used.

We agree that it is important to clarify not only that the working-heart mode is more physiological, but also why certain questions cannot be fully addressed using the Langendorff preparation. Our intention was not to imply that the Langendorff technique leads to false interpretations, but rather that its isovolumic nature and lack of change of afterload inherently limit the physiological inferences that can be drawn. Hence, the working-heart allows investigators to answer different types of questions that the Langendorff cannot. We have added this sentence to the Introduction (lines 98 to 100).

Line 303: Under these conditions, the heart continued to beat for up to five hours. This is indeed important for many experimental setups. Please give more information about the stability of isolated heart preparations in the Langendorff mode and the working heart mode.

We thank the reviewer for this valuable suggestion. In response, we have added a dedicated discussion comparing the stability of isolated heart preparations in both the Langendorff and working-heart modes (see lines 493 to 500).

Line 315: Based on experiments with isolated frog 315 hearts, he developed mathematical models to investigate how changes in vascular elasticity 316 and resistance shaped the arterial pulse(Frank, 1990...).

I am afraid that Otto Frank is not alive in 1990! He can hardly developed such models in 1990!

Thank you for pointing this out. We agree that the previous wording unintentionally implied that Otto Frank developed his models in 1990. We have revised the sentence to clarify that the 1990 citations refer to modern analyses and summarises Frank's original work from the 19th century (lines 359 to 366). The corrected text now makes clear that the modelling and experimental developments were performed by Frank in the 1890s, with later authors providing contemporary interpretation.

Dear Dr Han,

Re: JP-TR-2026-291198R1 "**Presentation of preload and afterload to isolated heart preparations**" by Esau Mhandu, Toan Pham, Kenneth Tran, Andrew Taberner, and June-Chiew Han

We are pleased to tell you that your paper has been accepted for publication in The Journal of Physiology.

The last Word (or similar) version of the manuscript provided will be used by the Production Editor to prepare your proof. When this is ready you will receive an email containing a link to Wiley's Online Proofing System. The proof should be thoroughly checked and corrected as promptly as possible.

Authors should note that it is too late at this point to offer corrections prior to proofing. Major corrections at proof stage, such as changes to figures, will be referred to the Editors for approval before they can be incorporated. Only minor changes, such as to style and consistency, should be made at proof stage. Changes that need to be made after proof stage will usually require a formal correction notice.

All queries at proof stage should be sent to: TJP@wiley.com.

Are you on Twitter? Once your paper is online, why not share your achievement with your followers? Please tag The Journal (@jphysiol) in any tweets and we will share your accepted paper with our 30,000 followers!

TRANSPARENT PEER REVIEW POLICY: To improve the transparency of its peer review process, The Journal of Physiology publishes online as supporting information the peer review history of all articles accepted for publication. Readers will have access to decision letters, including Editors' comments and referee reports, for each version of the manuscript, as well as any author responses to peer review comments. Referees can decide whether or not they wish to be named on the peer review history document.

Yours sincerely,

Laura Bennet
Senior Editor
The Journal of Physiology

P.S. - You can help your research get the attention it deserves! Check out Wiley's free Promotion Guide for best-practice recommendations for promoting your work at www.wileyauthors.com/eeo/guide. You can learn more about Wiley Editing Services which offers professional video, design, and writing services to create shareable video abstracts, infographics, conference posters, lay summaries, and research news stories for your research at www.wileyauthors.com/eeo/promotion.

• IMPORTANT NOTICE ABOUT OPEN ACCESS: To assist authors whose funding agencies mandate immediate public access to published research findings, The Journal of Physiology allows authors to pay an Open Access (OA) fee to have their papers made freely available immediately on publication.

The Corresponding Author will receive an email from Wiley with details on how to register or log in to Wiley Authors where you will be able to place an order.

You can check if your funder or institution has a Wiley Open Access Account here:
https://authors.wiley.com/author-resources/Journal-Authors/open-access/author-compliance-tool.html

MEMBER DISCOUNT: Corresponding authors who are Members of the Physiological Society are eligible for a 20% discount on the cost of the Article Processing Charge (APC). To claim this discount, the Corresponding Author will need to log in to the Physiological Society Membership portal and retrieve the discount code (https://www.physoc.org/journals-and-media/journals/journal-discount-for-members/). This code should then be quoted in the billing section of Wiley Authors when selecting the open access option.

EDITOR COMMENTS

Reviewing Editor:

The reviewers are both satisfied with this revision, thank you for providing a timely response.

REFEREE COMMENTS

Referee #1:

The authors nicely addressed previous comments and improved the design of the review, which elevates impact/influence.

Referee #2:

I thank the authors for the clarifications.
